# Recommendations of the Brazilian Society of Nephrology for hemodiafiltration in maintenance dialysis therapy

**DOI:** 10.1590/2175-8239-JBN-2026-0161en

**Published:** 2026-10-05

**Authors:** Fernanda Salomão Gorayeb-Polacchini, Rosilene Motta Elias, Ana Lydia Lédo de Castro Ribeiro Cabeça, Dirceu Reis da Silva, Elizabeth Machado, Farid Samaan, Isadora Cartaxo de Sousa Calvo, José Carolino Divino, Kalyanne Cabral de Paula do Ó, Maria Gabriela Motta Guimarães, Maria Goretti Moreira Guimarães Penido, Patrícia Ferreira Abreu, Stenio Barbosa de Freitas, Vera Hermina Kalika Koch, José A. Moura-Neto

**Affiliations:** 1 Brazilian Society of Nephrology, São Paulo, SP, Brazil.; 2 Hospital de Base-FUNFARME, São José do Rio Preto, SP, Brazil.; 3 Universidade de São Paulo, São Paulo, SP, Brazil.; 4 Universidade Nove de Julho, São Paulo, SP, Brazil.; 5 Fundação Hospital de Clínicas Gaspar Vianna, Belém, PA, Brazil.; 6 Hospital de Clínicas de Porto Alegre, Porto Alegre, RS, Brazil.; 7 Instituto de Doenças Renais, Porto Alegre, RS, Brazil.; 8 Universidade do Vale do Rio dos Sinos, São Leopoldo, RS, Brazil.; 9 Secretaria de Saúde do Estado de São Paulo, São Paulo, SP, Brazil.; 10 Instituto Dante Pazzanese de Cardiologia, São Paulo, SP, Brazil.; 11 Karolinska Institutet, Department of Clinical Science, Intervention and Technology, Division of Renal Medicine, Stockholm, Sweden.; 12 VIVATUM PD AG, Eschenbach, Switzerland.; 13 Vicenza International Institute of Kidney Research, Vicenza, Italia.; 14 Universidade Federal da Bahia, Programa de Pós-Graduação em Ciências da Saúde, Salvador, BA, Brazil.; 15 Santa Casa de Belo Horizonte, Unidade de Nefrologia Pediátrica, Belo Horizonte, MG, Brazil.; 16 Universidade Federal de Minas Gerais, Faculdade de Medicina, Belo Horizonte, MG, Brazil.; 17 Universidade Federal de São Paulo, São Paulo, SP, Brazil.; 18 Hospital São João de Deus, Divinópolis, MG, Brazil.; 19 Universidade de São Paulo, Faculdade de Medicina, Hospital das Clínicas, Instituto da Criança, São Paulo, SP, Brazil.; 20 Faculdade Bahiana de Medicina e Saúde Pública, Salvador, BA, Brazil.

**Keywords:** Hemodiafiltration, Online Hemodiafiltration, Renal Insufficiency, Chronic, Renal Replacement Therapy, Quality Control, Consensus

## Abstract

Hemodiafiltration (HDF) combines diffusive and convective clearance, enhancing the removal of uremic toxins, particularly middle molecules, compared with conventional hemodialysis (HD). Recent evidence, including meta-analyses and the CONVINCE trial, suggests that high-volume HDF is associated with improved clinical outcomes, particularly when adequate convective volumes are achieved. In Brazil, the expansion of this modality has been accompanied by studies demonstrating its feasibility, safety, and applicability across different clinical settings. This position statement from the Brazilian Society of Nephrology (SBN), educational rather than normative in nature, provides recommendations based on the best available evidence and expert consensus regarding the use of HDF in maintenance dialysis in Brazil. The document addresses patient selection, minimum prescription parameters, operational targets, technical and safety requirements, as well as ethical, regulatory, and economic aspects, with the aim of supporting clinical and managerial decision-making, reducing practice variability, and promoting safer, higher-quality dialysis care.

## INTRODUCTION

Hemodiafiltration (HDF) represents an important evolution in the treatment of chronic kidney disease (CKD), combining the mechanisms of diffusion and convection to increase the clearance of uremic toxins of different molecular weights and overcome limitations of therapies in which a single solute transport mechanism predominates, such as hemodialysis (HD), hemofiltration (HF), or peritoneal dialysis (PD)^1,2^.

The conceptual evolution of HDF resulted from the recognition that clearance centered exclusively on small molecules was not sufficient to cover the entire complexity of the uremic syndrome. This understanding fostered interest in strategies capable of removing higher-molecular-weight molecules, especially by means of convective transport^3^.

This concept motivated Lee Henderson and colleagues to investigate convective transport as a therapeutic strategy to expand the removal of these substances^4^. In 1967, the group published the first article on the use of diafiltration as a dialysis method^4^. Subsequently, Henderson’s clinical work was fundamental for HDF to be understood as the integration of hemodialysis and hemofiltration, bringing together diffusive efficiency in the removal of small solutes and convective gain in the removal of middle molecules^5,6^. However, the most significant experience was conducted by Leber in 1978, in Germany^7^. Unlike the very small series published up to that point, it describes a structured clinical experience, involving patients on regular treatment, focusing not only on solute removal criteria but also on clinical outcomes^7^.

Although the concept of HDF was formally proposed in the late 1970s, its wider clinical application became possible with the development of high-permeability (high-flux) synthetic membranes, particularly polysulfone membranes. These membranes have high hydraulic permeability, a sieving coefficient for β2-microglobulin greater than 0.6, and less activation of the complement system when compared with conventional cellulose membranes^8,9^.

The most transformative technological advancement occurred with the development of online HDF, in which the replacement solution is produced in real time from the ultrapure dialysate through double filtration systems. This innovation has simplified the process, increased microbiological safety, and made the technique economically viable by eliminating the need for sterile bags of previously prepared replacement solution^10,11^.

From a clinical point of view, the pathophysiological mechanisms that support the potential benefits of HDF are related to the increased removal of uremic toxins, including middle molecules, protein-bound toxins, inflammatory cytokines, and advanced glycation products, substances associated with increased cardiovascular risk, erythropoietin resistance, and higher mortality in patients with advanced CKD undergoing dialysis (CKD-5D)^2,12,13^.

In subsequent decades, HDF adoption has occurred heterogeneously across different regions of the world. Data from the Japanese registry show that 63.3% of patients were on HDF in 2024^14^. Data from the 2024 European registry show wide international variation in the use of HDF. France has substantial use of the modality, Italy shows an intermediate profile, and Spain shows marked regional variation^15^. Similarly, Nordic countries also have high adoption of the technique: in Sweden, about 50–55% of patients on treatment receive online HDF. This global heterogeneity has been documented in international analyses of nephrology practice, which highlight the role of structural, economic, and regulatory factors in the adoption of different therapeutic modalities across countries^16^.

In the United States (U.S.), regulatory barriers related to the safe production of the replacement solution online delayed the implementation of the technique, with the approval of HDF systems by the Food and Drug Administration (FDA) obtained only recently^17^.

In Brazil, HDF was incorporated into the List of Procedures of the National Supplementary Health Agency (ANS) in 2021, followed by a progressive increase in its use among patients with supplementary health insurance^18^. Data from the Annual Dialysis Survey of the Brazilian Society of Nephrology (SBN) show a significant increase in the use of HDF in Brazil, from 1.8% in 2021 to 7.1% in 2024, surpassing the prevalence of PD (5.6%)^19^. Data from 2025 indicate further progression, with 8.5% of patients on HDF versus 4.2% on PD; in the supplementary health sector, 40.5% of dialysis patients undergo HDF^20^.

In this way, HDF represents the consolidation of more than five decades of conceptual, technological, and clinical development in dialysis therapy. Contemporary scientific evidence, particularly the results of the CONVINCE trial with high-volume convective HDF^21^, positions this modality as an important therapeutic strategy for patients with CKD on dialysis (CKD-5D), with the potential to establish itself as a relevant component of dialysis care worlwide^22^. In Brazil, the experience demonstrates the technical feasibility, safety, and reproducibility of the benefits described internationally, positioning the country as an active participant on the global stage of this advanced dialysis modality^23,24,25^.

### HDF modalities

HDF combines diffusive and convective solute transport. Since convective removal involves the extraction of large volumes of ultrafiltrate, it is necessary to infuse a sterile replacement solution into the extracorporeal circuit, in a volume proportional to the prescribed convective dose^26^. According to the infusion site of the replacement solution, four modalities are described: post-dilutional, pre-dilutional, mixed dilution, and mid-dilution^27^. The last two are rarely used in clinical practice, and the literature regarding their application and clinical benefits is more limited^28^. Thus, the modalities of greatest practical relevance are post-dilutional and pre-dilutional^29^.

In HDF, the convective volume corresponds to the total volume generated by convection over the course of the session and is operationally defined by the sum of the replacement volume and the net ultrafiltration volume. The replacement volume, in turn, corresponds to the volume reinfused into the patient. In the online HDF systems most commonly used in current practice, the convective volume is determined as a function of blood flow (Qb), filtration fraction (FF), and treatment time.

The most commonly used modality in Europe and Brazil is post-dilutional^21,23,30,31,32,33^. In this strategy, the replacement solution is infused after the dialyzer, preserving higher concentrations of solutes inside the dialyzer and providing greater purification efficiency per unit of convective volume, especially for small solutes and middle molecules. On the other hand, the progressive intradialyzer hemoconcentration favors increased blood viscosity, protein deposition on the membrane, increased transmembrane pressure (TMP), and a greater risk of system coagulation. For this reason, the FF is usually limited to 25% of the Qb and can reach values close to 30% under appropriate technical conditions^10,26,30^. FF should be understood as the proportion of fluid removed from plasma during its passage through the dialyzer and not only from whole blood, which is particularly relevant in patients with higher hematocrit, in whom hemoconcentration, increased TPM, and limitations in convective dose delivery may be more pronounced. For high-volume post-dilutional HDF, a high Qb, typically greater than 350 mL/min, and a well-functioning vascular access are usually required^27^. Under these conditions, high convective volumes are more easily achieved in patients with arteriovenous fistulas (AVF) compared with central venous catheters. The convective dose in this modality is mainly influenced by session time, Qb, and FF, which is one of the main limiting factors^10^.

In the pre-dilutional modality, the replacement solution is infused before the dialyzer, promoting the hemodilution of the blood entering the dialyzer. This configuration reduces intradialyzer hemoconcentration, minimizes protein deposition on the membrane, and attenuates the increase in TPM, facilitating the delivery of therapy even under conditions of lower Qb. Conversely, since dilution reduces the concentration of solutes at the entrance of the dialyzer, the depurative efficiency per liter of replacement solution is lower than in the post-dilutional modality, requiring a larger replacement volume to obtain an equivalent convective dose. In Japan, where lower blood flows, around 250 mL/min, and high-flux dialyzers are usually used^32^, the pre-dilutional modality is widely used. In this modality, the absence of significant hemoconcentration reduces the limitation imposed by FF, allowing the convective dose to be achieved with less dependence on elevated Qb, although at the cost of greater consumption of replacement volume.

In summary, post-dilutional HDF has higher depurative efficiency per unit of convective volume, but it depends on more favorable technical conditions, especially high Qb and well-functioning vascular access. Pre-dilutional HDF, on the other hand, is less efficient per liter of replacement volume, but offers greater operational stability, lower intradialyzer hemoconcentration, and greater feasibility in patients with vascular access or Qb limitations. In any case, clinical tolerance and overall effectiveness can be similar between the two modalities, provided that the technical particularities of each strategy are respected^31^. Thus, the choice of modality should be individualized, considering the therapeutic objectives, the performance of the vascular access, the ability to achieve the target convective volume, and the characteristics of the membrane used.

### HDF scientific evidence

#### Reduced mortality

The evidence on HDF in patients with CKD-5D has evolved from randomized controlled trials (RCTs) with initially heterogeneous results to more consistent meta-analyses and real-world studies. While the CONTRAST trial and the Turkish study did not demonstrate a significant reduction in mortality in the primary analysis, ESHOL showed a reduction in overall mortality with post-dilutional high-volume convective HDF, suggesting that the benefit is related to adequate convective dose delivery^34,35,36^.

This finding was reinforced by subsequent meta-analyses. An individual patient data meta-analysis published in 2016 demonstrated a reduction in overall and cardiovascular mortality with HDF, with a more evident effect when the convective volume exceeded 23 L/session^37^. Consistently, the CONVINCE study confirmed a 23% reduction in all-cause mortality with high-volume HDF, as well as lower mortality from infection, compared with high-flux HD^21^.

More recent meta-analyses have consolidated these findings, confirming a reduction in overall, cardiovascular, and infection-related mortality with HDF, as well as a dose-response relationship between higher convective volume and a lower risk of death, especially with volumes ≥ 23 L/session^38,39^. In Brazil, these results were corroborated by a multicenter retrospective cohort, in which high-volume HDF was associated with a reduction in overall and cardiovascular/cerebrovascular mortality^40^, a result similar to that of other observational studies^41,42^.

#### Reduced infection and hospitalization rates

Considering the applicability of HDF in clinical practice outside the context of an RCT, there is evidence to support its real-world benefit. A large retrospective international study of 71,669 patients compared high-volume HDF with high-flow HD and showed reduced rates of hospitalization [incidence rate ratio (IRR) 0.80; 95% CI 0.79–0.82] and fewer hospitalization days (IRR 0.80; 95% CI 0.78–0.82), with consistency across several clinical and dialysis subgroups^43^.

#### Benefit for vascular access

In the area of vascular access, a pillar of extracorporeal therapies and, in the case of HDF, one of the main variables for achieving an adequate convective volume, a recent Japanese cohort study demonstrated that HDF was associated with greater primary patency of the fistula at 24 months (74.0% vs. 48.7% in HD) and with a significant reduction in primary patency loss (HR 0.30; 95% CI 0.16–0.56), in addition to a lower need for endovascular interventions, even after adjusting for confounding variables^44^.

#### Metabolic benefit

Regarding the impact of HDF on laboratory markers, a significant reduction in serum phosphate has been observed, especially when high convective volumes are reached, which may decrease the need for phosphorus binders^45,46^. Regarding protein-bound uremic toxins, such as indoxyl sulfate and p-cresyl sulfate, HDF has been shown to be more effective in reducing pre-dialysis concentrations of these markers, with a more pronounced effect for p-cresyl sulfate in patients who reach convective volumes greater than 27.5 L^46^.

Post-dilutional HDF removes even more beta-2-microglobulin (β2M) than high-flux HD^47,48^. The study by Armenta-Álvarez et al. demonstrated that HDF provides a higher rate of β2M removal compared with high-flux HD while maintaining an acceptable profile of dialytic albumin loss under appropriate prescription parameters. The convective component of HDF proved to be decisive in its superior clearance of molecules in the intermediate molecular weight range (~11.8 kDa), reinforcing the pathophysiological plausibility of this benefit^49^.

#### Improvement in symptoms and quality of life

HDF has been associated with better treatment tolerance and improvement in patient-reported symptoms when compared with high-flux HD. The better hemodynamic tolerance of HDF was clearly demonstrated in the study by Morena et al., in which elderly patients had better tolerance to treatment when compared with elderly patients receiving high-flux HD, with fewer episodes of vital sign changes, a significant reduction in symptoms during the intradialytic period, and better outcomes related to quality of life^50^.

In the CONVINCE trial, the positive effects of HDF on quality of life were also observed^51^. In a study conducted by Aichi et al., evaluating patients in an HD program who had low levels of health-related quality of life (HRQoL), conversion to HDF was associated with a significant improvement in quality-of-life scores and nutritional status^52^. In a systematic review and meta-analysis published by Bossola et al., which assessed the impact of HDF on quality of life, fatigue, and post-dialysis recovery time, the modality was associated with significant improvements in these outcomes when compared with conventional HD^34^. The pooled analysis showed a reduction in fatigue and a shorter recovery time after the sessions, in addition to improvements across HRQoL domains, suggesting a consistent clinical benefit^53^.

Thus, the growing incorporation of HDF into clinical practice imposes the need for an evidence-based position statement that guides its indication, its technical requirements, and its application in different care settings.

This is an institutional document of an educational and non-normative nature. The objective of this position statement is to provide SBN recommendations, based on the best available evidence and expert consensus, for the use of HDF in maintenance dialysis in Brazil. It seeks to standardize concepts, reduce the variability of clinical practice, and support clinical and managerial decision-making, including the selection and indication of patients; minimum prescription parameters and operational goals; technical and safety requirements; and ethical, regulatory, financial, and cost-effectiveness aspects, with a focus on the quality and safety of patient care.

## METHODS

This document consists of official recommendations from the SBN and was prepared by authors who are members of the National Board of Directors, the Department of Dialysis, the Department of Pediatric Nephrology, and the SBN Patient Committee, as well as by an external guest with extensive experience in HDF on the international stage.

The elaboration process involved structured discussions among the authors, based on the available scientific literature and international guidelines. In a subsequent stage, the document was submitted for review by the active SBN Regional Chapters and made available for public consultation on the SBN website for a period of ten days. The consultation was publicized on the SBN website and social media, allowing the participation of professionals, healthcare services, and other interested parties. The form made available during the public consultation presented the document’s recommendations and allowed for structured responses through the options “I agree with the document”, “I partially agree with the document”, and “I disagree with the document”, in addition to an open field for comments, justifications, and suggestions for modification. The contributions received were analyzed and systematized by the group, with the incorporation of pertinent suggestions and the justification for those not incorporated, with the objective of maximizing transparency, legitimacy, and adherence to the country’s needs.

After consolidation following the public consultation, the document underwent editorial review and final approval by those responsible, resulting in the definitive version of the recommendations.

### Results of the public consultation

In the public consultation, 124 contributions were received, of which 118 (95.2%) expressed full agreement, 5 (4.0%) partial agreement, and 1 (0.8%) disagreement. Of the 124 contributions, five items were suggested for modification, of which 4 (80%) were incorporated into the final version of the document. Of the participants in the public consultation, 74 (59.7%) were nephrologists, 30 (24.2%) patients, 14 (11.3%) health professionals, and 5 (4.0%) other interested parties. The contributions submitted during the public consultation, together with the author’s respective considerations, are available upon request to the SBN and with reasonable justification. The names of the authors of the contributions submitted during the public consultation were removed following the recommendation of the SBN Legal Department.

## DISCUSSION

### Patient eligibility for HDF therapy

As previously discussed, HDF is associated with reduced mortality, with potential favorable effects also on hospitalization and quality of life. It can be indicated for a broad range of patients with CKD-5D, provided there is shared decision-making and technical conditions that allow it to be performed safely^38,39,40,43^.

Although there are no universally established absolute contraindications, HDF depends on appropriate clinical conditions, adequate infrastructure, and the ability to maintain effective convective delivery. In addition, its effect compared with conventional hemodialysis is not homogeneous and may vary according to the patient’s clinical profile, the characteristics of the treatment, and the feasibility of achieving the desired therapeutic parameters^26^.

This heterogeneity was observed in a predictive model derived from the major comparative studies between HD and HDF, in which a trend toward greater survival gains was identified among younger patients with a lower burden of comorbidities, higher levels of albumin and creatinine, and lower levels of C-reactive protein (CRP)^21^.

In general, based on clinical plausibility and accumulated experience, the benefits of HDF may be more evident: (1) in patients with lower residual renal function—who may benefit from broadening the spectrum of uremic toxins removed by a technique that combines diffusion and convection; and (2) in younger individuals, with a longer life expectancy and no prospect of kidney transplantation—which may be associated with greater cumulative exposure to the therapy and its potential benefits. In resource-limited settings, these profiles may be considered priorities for the incorporation of HDF^26^.

Thus, the indication for HDF should be individualized, considering the potential clinical benefit, technical feasibility, patient preference, and the structural resources available at the service. Rigid and universally predefined criteria for patient selection are not proposed, and this decision should remain at the discretion of the attending physician, based on an individualized clinical evaluation.

### Limitations of vascular access or hemoconcentration

A fundamental requirement for the success of HDF is adequate vascular access in the post-dilutional HDF modality. To achieve a convective volume of ≥ 23 L, vascular access capable of providing a Qb greater than 350 mL/min is required^27,30^. Hemoconcentration is also relevant because when hematocrit is > 35%, the blood becomes more viscous, increasing the FF and potentially compromising adequate delivery of the therapy^2^.

### Frail elderly patients with advanced comorbidities

In addition to survival, it is important to highlight the evidence supporting patient-centered outcomes. In the elderly, there is better intradialytic tolerance and a tendency toward lower hospitalization with the use of HDF^50^. In the specific analysis of quality of life in the CONVINCE trial, patients on HDF showed a slower decline in the physical health (*p* = 0.035) and cognitive function (*p* = 0.031) components, with an overall improvement in health-related quality of life at 30 months (*p* = 0.006), reinforcing a clinically relevant impact on the patient-reported outcomes^51^. When quality of life is prioritized, HDF becomes a modality of interest in the frail elderly population.

### Pregnant women

To date, however, there are no specific studies evaluating the use of HDF during pregnancy. The available literature emphasizes the intensification of conventional HD—with increased frequency and duration of sessions—a strategy associated with better maternal and fetal outcomes^54^. However, some international experiences and position statements from scientific societies suggest a potential benefit of the technique, based on physiological plausibility, particularly due to greater depurative efficiency and better hemodynamic tolerance. In this context, the Chilean Society of Nephrology recommends HDF as the preferred modality for pregnant women on dialysis. Even so, the absence of specific evidence limits the generalization of this recommendation, and the choice of modality should be individualized^55^.

### Children

HDF in the pediatric population began in the 1970s in Strasbourg, France, with Fischbach and colleagues, initially using the pre-dilutional modality. Unlike in adults, the prescription of HDF in pediatrics should be normalized to body surface area. The recommended convective volume target is 13–15 L/m^2^ per session in the post-dilutional modality. To achieve these volumes, it is essential to optimize the Qb to 5–8 mL/min/kg or 150–250 mL/min/m^2^. FF should be maintained between 30% and 35% of the established Qb to avoid hemoconcentration and coagulation of the system^56^.

When HDF treatment is initiated, an initial Qb rate of 90–100 mL/min is suggested for the first few sessions, with increments of 10 mL/min/m^2^ per week, up to 200–250 mL/min/m^2^ as tolerated. A critical challenge is the limitation of equipment for young children. Currently, there are no HDF machines approved for patients under 10 kg. The Fresenius 6008 model, for example, is indicated for children weighing more than 10–12 kg, with the use of pediatric circuits, as specified in Charts 1 and 2 ^56^.

**Chart 1:** Specifications and requirements for pediatric HDF.

Technical category	Specifications and requirements for pediatric HDF
**Minimum weight**	**10 kg to 12 kg** (limit for the Fresenius 6008 machine with pediatric circuit). Other models require higher weights: **> 15 kg** (5008); **> 25 kg** (Baxter Artis Physio); **30 kg** (Dialog iQ). There are no machines validated for patients weighing < 10 kg
**Machine**	**Fresenius 6008** (currently the main machine available for pediatric use in this weight range) **Fresenius 5008/S** (being phased out) **Baxter Artis Physio** **Braun Dialog iQ**
**Dialyzer specifications**	**Sieving coefficient (**β2M)*:* > 0.6 **Surface area:** 100% and 120% of the child’s body surface area **Fiber diameter:** ≥ 200 *μm* (to minimize hemoconcentration)
**HDF blood lines**	**Specific pediatric circuits** (low-volume lines to reduce extracorporeal volume) and **disposable infusion/replacement lines** integrated into the online system
**Fluid type**	**Online replacement solution** **Quality**: ultrapure, sterile, and non-pyrogenic water **Purity:** < 0.1 CFU/mL and endotoxins < 0.03 EU/mL

**Chart 2:** Pediatric HDF with the 6008 system.

Technical category	Specifications for the 6008 CAREsystem paed
**Minimum Weight**	**10 kg to 12 kg** (safe operating limit when using the specific pediatric circuit)
**Machine**	Fresenius 6008 (successor model to the 5008, currently the only one available with validation for children in this weight range)
**Configuration**	**6008 CAREsystem Paed**: system that includes the low-volume pediatric extracorporeal circuit to minimize the risk of hemodynamic instability
**Dialyzer specifications**	**High-flux** KUF: > 20 mL/h/mmHg (ideally > 50 for maximum efficiency); permeability to β2M > 0.6 Surface area: 100% to 120% of the patient’s body surface area
**HDF blood lines**	**Integrated pediatric circuits:** low-dead-space blood lines and infusion lines for automated online replacement
**Fluid type**	**Online replacement solution (ultrapure water):** Standard: sterile and non-pyrogenic (internal filtration and backfiltration protected by endotoxin-retaining filters) Purity: < 0.1 CFU/mL and endotoxins < 0.03 EU/mL
**Infusion modality**	**Post-dilution**: preferred modality with the use of **AutoSub Plus™**, which automatically optimizes convective volume and filtration fraction in real time
**Points of attention in clinical practice**	Convective volume: the therapeutic goal should be 12 to 15 L/m^2^ per session to ensure benefits for growth and the reduction of inflammation Filtration fraction (FF): the 6008 system allows maintenance of a safe FF of up to 30–35%, preventing excessive hemoconcentration in the dialyzer BVM (blood volume monitor): monitors variations in relative blood volume during dialysis BTM (blood temperature monitor): monitors blood temperature and recirculation in the vascular access Online clearance monitor (OCM): monitors the effectiveness of dialysis (clearance) and the urea reduction ratio in real time Vascular access: the CAREsystem Paed requires blood flows between 5 and 8 mL/min/kg to achieve optimal convective performance

HDF has emerged as a potentially superior treatment modality to conventional HD in children and adolescents with CKD-5D, due to its ability to mitigate cardiovascular risk, provide better blood pressure control, reduce the concentration of inflammatory toxins, promote bone health, and, above all, improve quality of life, with shorter post-dialysis recovery time, greater physical fitness, and lower school absenteeism^56,57,58,59^.

Subclinical cardiovascular disease is prevalent in children and adolescents on dialysis. The 2019 3H multicenter observational study *(The HDF, Heart and Height Study)* ^60^ is the only multicenter pediatric analysis involving European and North American countries that compares HDF and HD. The 3H data suggest significant benefits of HDF over HD after one year of therapy. The study used the post-dilutional modality and demonstrated a significant increase in the carotid intima-media thickness (cIMT) Z-score in children on HD, whereas this parameter remained stable in the HDF group. In the propensity score analysis, HD was associated with a 0.47 increase in the annualized cIMT Z-score compared with HDF. The mean arterial pressure Z-score increased only in the HD group. The factors associated with higher cIMT and mean arterial pressure Z-scores were the HD group, a higher ultrafiltration rate, and higher levels of β2M. The HDF group had lower levels of β2M, parathyroid hormone, and high-sensitivity C-reactive protein^58,60,61^.

Growth retardation is an impactful complication in dialyzed children. Fischbach et al. were the first to demonstrate that growth retardation in children with CKD-5D could be reversed through daily HDF, with significant improvement in growth velocity and the height Z-score, which increased in the HDF group but remained stable in the HD group. This beneficial effect is attributed to the clearance of inflammatory cytokines and somatomedin inhibitors (such as β2-microglobulin), which helps reducing resistance to growth hormone. In addition, improvements in appetite and body mass index have been observed^62^.

HDF improves bone health by favoring an osteoanabolic profile. The ratio of markers of bone formation and resorption increases in children undergoing HDF, reaching levels comparable to those of healthy children. Another vital benefit is a 25% reduction in FGF23 levels, a toxin that, when present in excess, is linked to left ventricular hypertrophy and mortality. In contrast, children on HD show an increase of more than 100% in FGF23 levels over the course of a year^63,64^.

For adolescents and children, HDF translates into a more active life. These patients report fewer intradialytic symptoms, including reduced headache, vertigo, and cramps. In addition, these patients have a shorter post-dialysis recovery time, improved school attendance, and greater capacity for physical activity^65^.

It should also be added that HDF is well tolerated, with hemodynamic stability and fewer episodes of intradialytic hypotension; there is no significant difference in the loss of residual renal function compared with HD, nor does it seem to negatively affect serum albumin^63,64,65^.

In conclusion, high-volume HDF emerges as the preferred dialysis modality for children and adolescents, as it is associated with better clinical outcomes compared with conventional HD, including greater removal of middle molecules, reduction of the chronic inflammatory state, favorable impact on linear growth and bone health, greater hemodynamic stability and vascular protection, promoting better blood pressure control, better quality of life, and greater social participation.

SBN recommendation: HDF may be considered for patients with 5D-CKD, provided that there is shared decision-making, technical feasibility, adequate vascular access or a strategy compatible with the chosen modality, the possibility of achieving an effective convective dose, and adequate service infrastructure. In resource-limited scenarios, children, patients with intradialytic symptoms, those with lower residual renal function, a higher middle-molecule load, young patients with no prospect of kidney transplantation, frail older adults with worse tolerance to conventional HD, and patients with greater potential for clinical benefit can be considered priorities, without these criteria constituting mandatory or restrictive indications.

### Evidence for home, incremental, more frequent, or nocturnal HDF

To date, there are no robust studies that directly compare home HDF with HDF performed in a dialysis center. The literature addresses the following comparisons between modalities (HD vs. HDF) and settings (home vs. dialysis centers), as illustrated in Chart 3.

**Chart 3:** Comparative studies of HDF and HD in home and in-center settings.

Comparison	Available evidence	Type of studies	Key findings
**Home HDF vs. in-center HDF**	No	—	No randomized trials or large cohort studies directly comparing home and in-center HDF were identified.
**In-center HDF vs. in-center HD**	Yes	Clinical trials and meta-analyses	High-volume HDF associated with lower mortality and possibly fewer cardiovascular events.
**Home HD vs. in-center HD**	Yes	Observational cohorts, systematic reviews	Studies suggest better quality of life, greater flexibility, and, in some analysis, better survival with home hemodialysis.
**Location of treatment (home vs. center) across HDF studies**	No	—	HDF studies do not stratify outcomes by treatment setting.

Abbreviations – HD: hemodialysis; HDF: hemodiafiltration.

Although there are no studies directly comparing home HDF with that performed in a dialysis center, multiple lines of evidence demonstrate that high-volume in-center HDF is associated with lower mortality in Brazil^40,41,42^ and worldwide^21,34,35,36^. Thus, it is plausible to assume that part of the physiological benefits of HDF can also be observed in the home environment. However, this extrapolation should be interpreted with caution, since it still lacks confirmation by specific evidence.

We cannot yet conclude, due to an absolute lack of data, whether home HDF can provide an additional reduction in mortality compared with in-center HDF. More frequent dialysis can lead to better survival; however, there is no comparison in the literature between HDF and HD modalities with regard to more frequent dialysis or long nocturnal dialysis.

As for incremental HDF, there is no evidence of its superiority compared with incremental HD. However, the benefit of incremental HD^66^ is unlikely not to be observed with HDF as well. Regarding decremental HDF, to date, there have been no specific studies on the use of HDF in delayed graft function after kidney transplantation.

Regarding long nocturnal HDF performed on alternate days, a single-center observational study by Maduell F et al.^67^ showed good clinical tolerance and the absence of adverse symptoms, with improved control of calcium-phosphorus metabolism and blood pressure, and reduced use of phosphate binders and antihypertensive drugs. An important aspect is that 62% of the patients maintained professional activity, a finding similar to that observed in an observational study in Brazil^68^.

SBN recommendation: Home-based, incremental, more frequent, or nocturnal HDF should be considered only in services with compatible technical infrastructure, staff training, monitoring, care support, and safety measures. In the absence of robust direct evidence, extrapolation of the benefits of high-volume in-center HDF to these settings should be done with caution and with individualized prescribing.

### Suggested minimum parameters for HDF prescription in adults

The decision between conventional HD and high-volume HDF should be shared between the team, the patient, and the patient’s family members. It is essential that the patient understands the benefits, risks, and requirements of each method, thereby favoring treatment adherence, by aligning expectations, especially regarding session time, the need for adequate vascular access, and session attendance^69^.

#### Transition from HD to HDF (step-up approach)

The transition from HD to HDF should occur after prior optimization of HD, allowing for progressive adjustments, greater safety, and improved therapeutic efficacy^32,70,71^.

The following is recommended:

1. Use of high-flux dialyzers: ultrafiltration coefficient (KUF) > 50 mL/h/mmHg/m^2^ and a sieving coefficient for β2M > 0.6.
2. Four-hour sessions, three times a week, for most patients.
3. Adequate vascular access: Qb of 350–450 mL/min with 14–15-gauge (G) needles in a fistula or graft, or a ≥ 14 French (F) catheter in the post-dilutional modality^72^.
4. The prescription should consider the required net ultrafiltration volume, seeking hemodynamic stability and clinical tolerance to the procedure.
5. Progressive prescription: start with a Qb of 250 mL/min for 15 days, increase to 300 mL/min for another 15 days, and subsequently reach the maximum effective level. This strategy may not be applicable to patients transitioning from HD, who already have a high, previously well-tolerated Qb. Start with a convective volume of 15–18 L/session, with a gradual increase in 2 and 3 weeks, according to clinical tolerance.

#### HDF prescription components^2,26^

The prescription must include well-defined minimum technical and operational parameters:

– The post-dilutional modality is the most studied and generally preferred, as it accounts for most of the available evidence. It requires a smaller replacement volume, with lower resource consumption and greater operational sustainability. The pre-dilutional modality can be considered in situations of Qb limitation (<350 mL/min) or a higher risk of hemoconcentration in the filter, such as in patients with high hematocrit, the need for a high net ultrafiltration volume, relative hypovolemia, recurrent intradialytic hypotension, low blood flow, or limited vascular access.
– AVF is the access of choice, followed by graft and tunneled catheter.
– Use 14–15G needles to achieve Qb > 350 mL/min; 16G can be used in selected cases of Qb < 350 mL/min in the pre-dilutional modality.
– Inadequate flow or recirculation of the vascular access contraindicates post-dilutional high-volume HDF. A Qb of 350–450 mL/min (post) and 200–450 mL/min (pre) is recommended.
– Minimum duration of four hours, three times a week, for most patients.
– Dialysate composition similar to that of HD, with a flow rate of 400–500 mL/min. In modern online HDF systems, the dialysate flow (Qd) can be adjusted relative to the Qb and the replacement flow. When maintained at around 20% above the Qb, it can preserve the clearance of small solutes and favor greater delivery of the convective dose, without a proportional increase in ultrapure water consumption^2^. The routine use of dialysate without glucose or with a potassium concentration below 2.0 mEq/L is not recommended, due to the higher risk of adverse events, except in exceptional situations and with strict monitoring.
– In the post-dilutional modality, maintain an FF ≤ 30% of the Qb to avoid hemoconcentration, albumin loss, and coagulation of the circuit^73^.
– Convective flow of 80–160 mL/min in the post-dilutional modality and 160–300 mL/min in the pre-dilutional modality, with a minimum convective volume of ≥ 23 L/session or ≥ 70 L/week in the post-dilutional modality, and of ≥ 46 L/session or ≥ 140 L/week in the pre-dilutional modality.
– Use dialyzers with KUF > 50 mL/h/mmHg/m^2^, a sieving coefficient for β2M > 0.6, low albumin permeability (sieving < 0.001), and a surface area of 1.5–2.2 m^2^.
– Follow the institutional anticoagulation protocol, with possible adjustments in the post-dilutional to avoid coagulation of the system.

Chart 4 summarizes the minimum recommendations for HDF prescription^10,74,75^.

**Chart 4:** Minimum recommendations for high-volume online HDF prescribing.

Vascular access	AVF (1st option)/graft/ tunneled catheter; avoid high-volume HDF if inadequate flow/recirculation.
**Needle**	14 or 15G → Qb > 350 mL/min; 16G → Qb of 300 to 350 mL/min (selected cases).
**Blood flow (Qb)**	Post: 350–450 mL/min; Pre: 200–450 mL/min.
**Dialysate flow (Qd)**	400–500 mL/min.
**Time**	≥ 4 h/session (12–15 h/week).
**Filtration fraction (FF)** (ratio of convective volume to Qb)	≤ 30% (ideal ≤ 25%) Post-dilutional.
**Modality**	Post-dilutional (most studied); consider pre-dilutional modality if Qb < 350 mL/min.
**Convective flow**	Post: 80–160 mL/min; Pre: 160–300 mL/min.
**Convective volume** (sum of replacement volume and ultrafiltration volume)	Post: ≥ 23 L/session or ≥ 70 L/week; Pre: ≥ 46 L/session or ≥ 140 L/week.
**HDF dialyzer type**	KUF > 50 e SC for β2M > 0.6; surface area of 1.5 to 2.2 m^2^ in adults; SC for albumin < 0.001; KoA for urea > 600 and low internal resistance to blood flow.
**Anticoagulation**	Heparin (UFH/LMWH) per protocol; may require adjustment in the post-dilutional modality.
**Goals**	Convective volume ≥ 23 L/session; spKt/V > 1.2.
**Monitoring**	Monthly review of prescription and access.

Abbreviations – SC: sieving coefficient; UFH: unfractionated heparin; LMWH: low molecular weight heparin; K_UF_: ultrafiltration coefficient.

#### Operational goals

They include a convective volume of ≥ 23 L/session (post-dilutional), spKt/V > 1.2, reduced intradialytic hypotension, and fewer session interruptions^21^. Vascular access, circuit pressures^76^, and clinical and laboratory parameters, such as nutritional status, electrolytes, anemia, tolerance to the procedure, and quality of life, should be monitored^77,78^.

Although the convective volume of ≥ 23 L/session is a practical goal in post-dilutional HDF, this target may be insufficient in individuals with a larger body surface area (BSA); therefore, adjustment for BSA should be considered using the formula 23 L × BSA/1.73 m^2^ to avoid relative under delivery of the convective dose^2^. The use of larger-surface-area dialyzers can be considered as a strategy to achieve the convective volume target when clinically appropriate and provided that technical and safety conditions are met.

The prescription should be individualized, with monthly reassessment according to the clinical response, ensuring adequacy of the dose, frequency, treatment duration, and indication for HDF.

#### Minimum technical requirements and safety

To implement and maintain a high-volume online HDF, there are requirements that must be met, namely^26^:

1. Dialysate composed of ultrapure water.
2. Online HDF machines.
3. Strict quality control, which includes disinfection procedures related to the HDF machine and periodic replacement of sterilizing ultrafilters.
4. Suitable HDF dialyzer (described in Chart 4).
5. A team of professionals trained to perform HDF.
6. Adequate vascular access (described in Chart 4).
7. Good practices: monitor patient signs and symptoms, optimize anticoagulation, dialysis adequacy, and the management of quality indicators.

These requirements are described in greater detail below.

Water Treatment: Considering that the replacement solution needs to be infused into the patient during HDF (either pre- or post-dialyzer), it must be sterile, nonpyrogenic, and produced and monitored at an acceptable cost for the healthcare system^23,26,30^. In Brazil, ANVISA Resolution (RDC) No. 11/2014 establishes Good Practices for dialysis services^79^ and scientific societies have sought to express their views on the expected water quality standards for HDF^80,81^. In addition, ISO 23500-1:2019 and ISO 23500-3:2019 describe specific requirements for this process and the expected water quality for hemodepurative therapies^10,82,83^.

In the HDF machine, the replacement fluid, which is infused into the bloodline (pre- or post-HDF filter), is produced online through ultrafiltration of the ultrapure dialysate by a series of sterilizing filters (called ultrafilters). These ultrafilters have an endotoxin adsorption capacity of 1:1,000,000, and thus the interposition of two filters in sequence allows a retention of 1:10 retention^-12^, constituting what is conventionally called “cold sterilization”. The process requires that the ultrapure water meet international standards of microbiological and chemical quality, such as those defined by the International Organization for Standardization (ISO) and the Association for the Advancement of Medical Instrumentation (AAMI), with strict limits for bacteria (<0.1 CFU/mL) and endotoxins (<0.03 EU/mL). Subsequently, the ultrapure water is mixed with the concentrates to form the dialysate, which passes through validated ultrafilters to produce the sterile replacement fluid^26,30,84,85^.

In HDF, the machine must be disinfected according to the manufacturer’s recommendations (including the ultrafilters), with an integrity test before each session and replacement of the filters at the recommended intervals^30^. Once the treated water has been confirmed to comply with the RDC No. 11/2014 standards and it has been ensured that the operation and monitoring of the HDF equipment are performed as per the manufacturer’s instructions, no additional routine testing is warranted^23^.

Monitoring in HDF should include essential parameters for controlling efficacy and safety in convective therapies, including Qb and convective volume. Achieving adequate convective volumes in each session is a central element of HDF efficacy^21,75^. The medical record should allow the systematic recording of these parameters to generate follow-up and management indicators, highlighting hemodynamic tolerance and adverse events^86^. ANVISA recommends patient safety practices in dialysis services, including protocols for water quality and monitoring, technology management, infection prevention, and collection/analysis of quality indicators, which also apply to HDF^87^.

Considering the importance of vascular access to achieve adequate convective volume, it should be evaluated longitudinally, and the use of an indicator based on the relationship between Qb and arterial pressure (AP) of the dialysis system (Qb/AP) is suggested, which reflects the patency of the vascular access, with a usual value of 2 mL/min/mmHg. A Qb/AP > 1.5 mL/min/mmHg is considered indicative of good flow, whereas flows between 0.8–1.5 mL/min/mmHg are considered poor, and the access would be in failure when Qb/AP < 0.8 mL/min/mmHg^26^. When the vascular access has Qb <350 mL/min, the cause of the low flow should be investigated and treated, and HDF can be conducted in the pre-dilutional modality.

The pre- and post-dilutional modalities and ultrafilters are exemplified in Figure 1.

**Figure 1:**
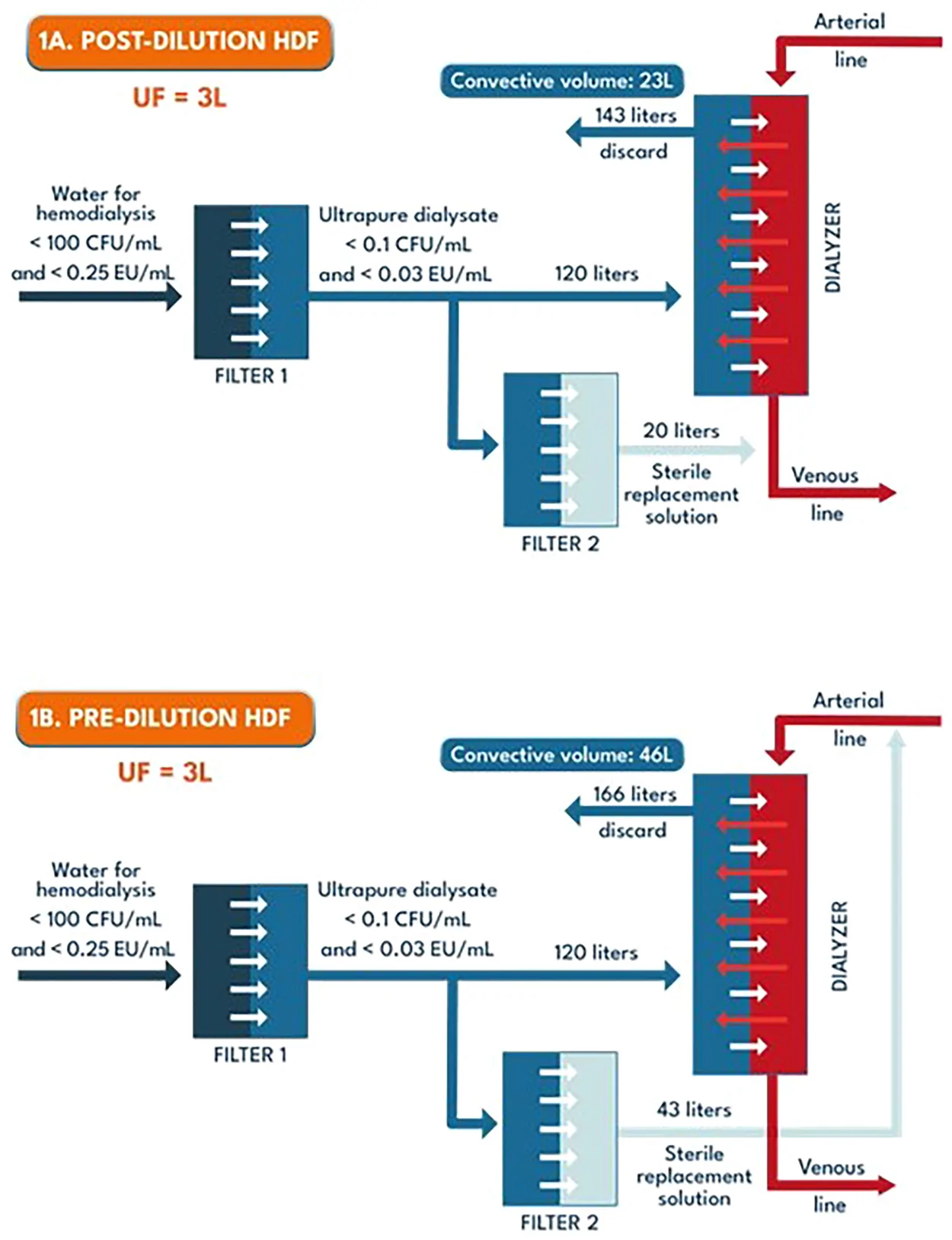
Comparative scheme of post- and pre-dilutional HDF, including filters, replacement solution, and ultrapure water.

Note – Adapted from Canziani MEF, et al.^23^

### Ethical, regulatory, financial, and cost-effectiveness aspects

The implementation of the recommendations in this document should consider ethical aspects, the capacity of health systems to offer dialysis, and, in the Brazilian context, possible regulatory adjustments and the improvement of reimbursement models. Therapeutic decisions should be based on clear information and shared decision-making; however, knowledge asymmetry between professionals and patients can compromise decision-making autonomy. In addition, the unavailability of certain therapeutic modalities can limit choices, aggravate communication failures, and restrict the autonomy of patients and professionals^88^.

The ethical factors of the community are related to the precepts of comprehensive care and social justice and, therefore, to the premise of using the public resources available for healthcare in the best possible way^89,90^. Microcosting studies indicate that the cost of the procedure per patient-month for HDF is higher than that for conventional HD, and the latter is higher than that for PD^91,92,93^. This difference can be less than 15% when considering only the financial costs required to produce inputs locally (cost), but the inclusion of other cost components (e.g., freight and import taxes) may change the commercial values (price)^94,95^.

In the case of renal replacement therapy (RRT) and the incorporation of high-cost technologies, a committee formed by members of the American Society of Nephrology, European Renal Association, European Dialysis and Transplant Association, and the International Society of Nephrology (ASN, ERA-EDTA, and ISN) has reinforced that public policies in Nephrology should be guided by the proportionality of actions and investments in prevention, conservative treatment, dialysis methods, and kidney transplantation^96,97,98^. This proportionality has not been observed in the provision of services by the Brazilian Unified Health System (SUS), as demonstrated in recent studies^99,100^.

About five years ago, HDF was included in the ANS List of Procedures^101^. However, this inclusion was not accompanied by a Technical Use Guideline (DUT), which means that private health plans should provide the procedure to beneficiaries, regardless of technical criteria, such as age, comorbidities, additional tests, among others.

In the case of RRT, in which the predominant reimbursement model in Brazil is fee-for-service, the absence of a DUT for HDF may contribute to inadequate denials by private health plans, as well as to unnecessary requests by healthcare providers^102,103^. The adoption of innovative reimbursement models could mitigate these events as they incorporate value-based healthcare elements into reimbursement (quality-of-care indicators, emergency room visits, hospitalizations, among others)^104,105^.

In Brazil, the healthcare system is characterized by the coexistence of the SUS – a public, universal system – and the supplementary sector. The SUS, which is chronically underfunded, is responsible for the exclusive care of about 75% of the population^106^. Considering nephrology care, studies have shown concerning differences between the clinical outcomes of Brazilians with and without supplementary health insurance^98,107^. The unequal incorporation of technologies between the public and supplementary health systems is a cause for concern. Although HDF has been included in the ANS List of Procedures since 2021, this modality has not yet been incorporated into the SUS. Currently, about 40.5% of dialysis patients in the supplementary sector undergo HDF, compared with approximately 0.5% in the SUS^20^, a scenario that may contribute to the expansion of inequities in the Brazilian health system, which is structured on the principle of equity.

Health technology assessment is fragmented in Brazil, with two main regulatory bodies: in the SUS, CONITEC (*Comissão Nacional de Incorporação de Tecnologias no SUS*) and, in the supplementary health sector, COSAÚDE (*Comissão de Atualização do Rol de Procedimentos e Eventos em Saúde Suplementar/ANS*). This duplicity constitutes a regulatory weakness that can contribute to lawsuits against the SUS and care inequities between Brazilians with and without health insurance. Australia, the United Kingdom, and Canada are international examples that have adopted a single health technology assessment agency^108^.

In line with international data, Brazilian studies have consistently shown HDF results superior to those of HD^23,40,41,42,109^, although complete economic evaluations have not yet been conducted (budgetary impact for the SUS, cost-effectiveness with national primary data, and use of methodologies that incorporate social determinants and regional differences)^110^.

### SBN recommendations for online high-volume HDF

The SBN’s key recommendations for online high-volume HDF are summarized in Chart 5.

**Chart 5:** Summary of SBN recommendations for high-volume online HDF.

1. Make a shared decision with patients and family members.
2. Consider HDF for all patients, as long as it is feasible to achieve an effective convective dose.
3. Prioritize, in resource-limited settings, patients with intradialytic symptoms, lower residual renal function, β2-microglobulin accumulation, young patients, and those with no prospect of kidney transplantation.
4. Some clinical profiles can be considered priorities for the incorporation of HDF, without this being a rigid or mandatory criterion for indication, which should remain individualized and under the responsibility of the attending physician.
5. Optimize high-flux HD beforehand and transition to HDF, with gradual increases in convective volume.
6. Prefer the post-dilutional modality; consider pre-dilutional when Qb <350 mL/min or when there is limited access/hemoconcentration.
7. Ensure four-hour HDF sessions, three times a week, for most patients.
8. Aim for a convective volume ≥ 23 L/session or ≥ 70 L/week in the post-dilutional modality. In the pre-dilutional modality, consider targets approximately twice as high, such as ≥ 46 L/session or ≥ 140 L/week. Prioritize AVF as the vascular access.
9. In the post-dilutional modality, aim for Qb of 350–450 mL/min (14–15G needles in AVF; tunneled catheter ≥ 14 F, when applicable).
10. Avoid high-volume HDF with inadequate flow/recirculation.
11. Use a dialyzer suitable for HDF.
12. Strictly follow the machine disinfection cycle schedule and ultrafilter replacement intervals.
13. Ensure ultrapure water dialysate.
14. Monitor indicators, including the convective volume rate.
15. Pediatrics: prescription normalized to body surface area; convective targets and adjusted FF limits; recognize equipment limitations in patients weighing < 10 kg.

## CONCLUSION

High-volume online HDF has become established as an advanced dialysis modality, with a consistent pathophysiological rationale and contemporary evidence of clinical benefit, especially when there is effective delivery of the convective dose (target ≥ 23 L/session in the post-dilutional modality). In Brazil, its recent expansion and national data on feasibility and safety reinforce the need for technical standardization, systematic monitoring, and quality governance. This SBN position statement proposes minimum prescription parameters, operational targets, and safety requirements while also acknowledging ethical, regulatory, and economic-financial challenges. Its adoption should be responsible, based on shared decision-making, adequate patient selection, and infrastructure capable of sustaining strict quality control and monitoring of care indicators, with a focus on safe, effective, and value-based care.

## Data Availability

No new data were generated or analyzed in this study.
