# Supplementary material for: Recommendations of the Brazilian Society of Nephrology for hemodiafiltration in maintenance dialysis therapy
Source: J Bras Nefrol. 2026 Oct 5;48(4):e20260161. doi: 10.1590/2175-8239-JBN-2026-0161en (PMC13660142; doi:10.1590/2175-8239-JBN-2026-0161en)
Supplement: Translation

# Translation: Recomendações da Sociedade Brasileira de Nefrologia para hemodiafiltração em terapia dialítica de manutenção

**DOI:** 10.1590/2175-8239-JBN-2026-0161pt

**Authors:** Fernanda Salomão Gorayeb-Polacchini, Rosilene Motta Elias, Ana Lydia Lédo de Castro Ribeiro Cabeça, Dirceu Reis da Silva, Elizabeth Machado, Farid Samaan, Isadora Cartaxo de Sousa Calvo, José Carolino Divino, Kalyanne Cabral de Paula do Ó, Maria Gabriela Motta Guimarães, Maria Goretti Moreira Guimarães Penido, Patrícia Ferreira Abreu, Stenio Barbosa de Freitas, Vera Hermina Kalika Koch, José A. Moura-Neto

**Affiliations:** 1 Brazilian Society of Nephrology, São Paulo, SP, Brasil.; 2 Hospital de Base-FUNFARME, São José do Rio Preto, SP, Brasil.; 3 Universidade de São Paulo, São Paulo, SP, Brasil.; 4 Universidade Nove de Julho, São Paulo, SP, Brasil.; 5 Fundação Hospital de Clínicas Gaspar Vianna, Belém, PA, Brasil.; 6 Hospital de Clínicas de Porto Alegre, Porto Alegre, RS, Brasil.; 7 Instituto de Doenças Renais, Porto Alegre, RS, Brasil.; 8 Universidade do Vale do Rio dos Sinos, São Leopoldo, RS, Brasil.; 9 Secretaria de Saúde do Estado de São Paulo, São Paulo, SP, Brasil.; 10 Instituto Dante Pazzanese de Cardiologia, São Paulo, SP, Brasil.; 11 Karolinska Institutet, Department of Clinical Science, Intervention and Technology, Division of Renal Medicine, Stockholm, Sweden.; 12 VIVATUM PD AG, Eschenbach, Switzerland.; 13 Vicenza International Institute of Kidney Research, Vicenza, Italia.; 14 Universidade Federal da Bahia, Programa de Pós-Graduação em Ciências da Saúde, Salvador, BA, Brasil.; 15 Santa Casa de Belo Horizonte, Unidade de Nefrologia Pediátrica, Belo Horizonte, MG, Brasil.; 16 Universidade Federal de Minas Gerais, Faculdade de Medicina, Belo Horizonte, MG, Brasil.; 17 Universidade Federal de São Paulo, São Paulo, SP, Brasil.; 18 Hospital São João de Deus, Divinópolis, MG, Brasil.; 19 Universidade de São Paulo, Faculdade de Medicina, Hospital das Clínicas, Instituto da Criança, São Paulo, SP, Brasil.; 20 Faculdade Bahiana de Medicina e Saúde Pública, Salvador, BA, Brasil.

**Keywords:** Hemodiafiltração, Hemodiafiltração *Online*, Insuficiência Renal Crônica, Terapia de Substituição Renal, Controle de Qualidade, Consenso.

## Abstract

A hemodiafiltração (HDF) combina difusão e convecção, ampliando a remoção de toxinas urêmicas, especialmente moléculas médias, em comparação à hemodiálise (HD) convencional. Evidências recentes, incluindo metanálises e o estudo CONVINCE, sugerem associação entre a HDF de alto volume e melhores desfechos clínicos, particularmente quando se atingem volumes convectivos adequados. No Brasil, a expansão do método tem sido acompanhada por estudos que demonstram sua viabilidade, segurança e aplicabilidade em diferentes cenários assistenciais. Este posicionamento da Sociedade Brasileira de Nefrologia (SBN), de caráter educativo e não normativo, apresenta recomendações baseadas nas melhores evidências disponíveis e em consenso de especialistas para o uso da HDF na diálise de manutenção no Brasil. O documento aborda seleção de pacientes, parâmetros mínimos de prescrição, metas operacionais, requisitos técnicos e de segurança, além de aspectos éticos, regulatórios e econômicos, com o objetivo de apoiar a prática clínica e gerencial, reduzir a variabilidade assistencial e promover um cuidado de maior qualidade e segurança.

## INTRODUÇÃO

A hemodiafiltração (HDF) representa uma evolução importante no tratamento da doença renal crônica (DRC), combinando os mecanismos de difusão e convecção para ampliar a depuração de toxinas urêmicas de diferentes pesos moleculares e superar limitações de terapias em que predomina um único mecanismo de transporte de solutos, como a hemodiálise (HD), a hemofiltração (HF) ou a diálise peritoneal (DP)^1,2^.

A evolução conceitual da HDF decorreu do reconhecimento de que a depuração centrada exclusivamente em pequenas moléculas não era suficiente para abranger toda a complexidade da síndrome urêmica. Esse entendimento favoreceu o interesse por estratégias capazes de remover também moléculas de maior peso molecular, especialmente por meio do transporte convectivo^3^.

Esse conceito motivou Lee Henderson e colaboradores a investigarem o transporte convectivo como estratégia terapêutica para ampliar a remoção dessas substâncias^4^. Em 1967, o grupo publicou o primeiro artigo sobre o uso da diafiltração como método dialítico^4^. Posteriormente, os trabalhos clínicos de Henderson foram fundamentais para a HDF ser compreendida como a integração entre hemodiálise e hemofiltração, reunindo a eficiência difusiva na remoção de pequenos solutos e o ganho convectivo na remoção de moléculas médias^5,6^. Entretanto, a experiência mais expressiva foi conduzida por Leber em 1978, na Alemanha^7^. Diferentemente das séries muito pequenas publicadas até aquele momento, o autor descreve uma experiência clínica estruturada, envolvendo pacientes em tratamento regular, com foco não apenas em critérios de remoção de solutos, mas também em desfechos clínicos^7^.

Embora o conceito de HDF tenha sido formalmente proposto no final da década de 1970, sua aplicação clínica mais ampla tornou-se possível com o desenvolvimento de membranas sintéticas de alta permeabilidade (alto fluxo), particularmente as membranas de polissulfona. Essas membranas apresentam elevada permeabilidade hidráulica, coeficiente de peneiramento (*sieving coefficient*) para β2-microglobulina superior a 0,6 e menor ativação do sistema complemento quando comparadas às membranas de celulose convencionais^8,9^.

O avanço tecnológico mais transformador ocorreu com o desenvolvimento da HDF *online*, na qual a solução de reposição é produzida em tempo real a partir do dialisato ultrapuro por meio de sistemas de dupla filtração. Essa inovação simplificou o processo, aumentou a segurança microbiológica e tornou a técnica economicamente viável ao eliminar a necessidade de bolsas estéreis de solução de reposição previamente preparadas^10,11^.

Do ponto de vista clínico, os mecanismos fisiopatológicos que sustentam os potenciais benefícios da HDF relacionam-se à remoção ampliada de toxinas urêmicas, incluindo moléculas médias, toxinas ligadas a proteínas, citocinas inflamatórias e produtos de glicação avançada, substâncias associadas ao aumento do risco cardiovascular, à resistência à eritropoietina e à maior mortalidade em pacientes com DRC avançada em diálise (DRC-5D)^2,12,13^.

Nas décadas subsequentes, a adoção da HDF ocorreu de forma heterogênea entre diferentes regiões do mundo. Dados do registro japonês demonstram que 63,3% dos pacientes estavam em HDF em 2024^14^. Os dados do registro europeu de 2024 evidenciam ampla variação internacional na utilização da HDF. A França apresenta utilização relevante da modalidade, a Itália mostra perfil intermediário e a Espanha exibe marcante variação regional^15^. De forma semelhante, países nórdicos também apresentam elevada adoção da técnica: na Suécia, cerca de 50–55% dos pacientes em tratamento recebem HDF *online*. Essa heterogeneidade global foi documentada em análises internacionais da prática nefrológica, que destacam o papel de fatores estruturais, econômicos e regulatórios na adoção de diferentes modalidades terapêuticas entre os países^16^.

Nos Estados Unidos (EUA), barreiras regulatórias relacionadas à produção segura da solução de reposição *online* retardaram a implementação da técnica, sendo a aprovação de sistemas de HDF pela *Food and Drug Administration* (FDA) obtida apenas recentemente^17^.

No Brasil, a incorporação da HDF ao Rol de Procedimentos da Agência Nacional de Saúde Suplementar (ANS) ocorreu em 2021, seguida por um crescimento progressivo de sua utilização entre pacientes da saúde suplementar^18^. Dados do Censo Anual de Diálise da Sociedade Brasileira de Nefrologia (SBN) evidenciam um aumento expressivo do uso de HDF no Brasil, de 1,8% em 2021 para 7,1% em 2024, superando a prevalência de DP (5,6%)^19^. Dados de 2025 indicam progressão adicional, com 8,5% dos pacientes em HDF *versus* 4,2% em DP; na saúde suplementar, 40,5% dos pacientes em diálise realizam HDF^20^.

Dessa forma, a HDF representa a consolidação de mais de cinco décadas de desenvolvimento conceitual, tecnológico e clínico na terapia dialítica. Evidências científicas contemporâneas, particularmente os resultados do ensaio CONVINCE com HDF de alto volume convectivo^21^, posicionam essa modalidade como uma importante estratégia terapêutica para pacientes com DRC em diálise (DRC-5D), com potencial para consolidar-se como componente relevante do cuidado dialítico em âmbito global^22^. No Brasil, a experiência demonstra viabilidade técnica, segurança e reprodutibilidade dos benefícios descritos internacionalmente, posicionando o país como participante ativo no cenário mundial dessa modalidade dialítica avançada^23–25^.

### Modalidades de HDF

A HDF combina transporte difusivo e convectivo de solutos. Como a remoção convectiva implica a extração de grandes volumes de ultrafiltrado, torna-se necessária a infusão de solução de substituição estéril no circuito extracorpóreo, em volume proporcional à dose convectiva prescrita^26^. De acordo com o local de infusão da solução de substituição, descrevem-se quatro modalidades: pós-dilucional, pré-dilucional, diluição mista e diluição média^27^. As duas últimas são pouco utilizadas na prática clínica e contam com literatura mais limitada quanto à sua aplicação e aos seus benefícios clínicos^28^. Assim, as modalidades de maior relevância prática são a pós-dilucional e a pré-dilucional^29^.

Na HDF, o volume convectivo corresponde ao volume total gerado por convecção ao longo da sessão, sendo operacionalmente definido pela soma do volume de substituição com o volume líquido de ultrafiltração. O volume de substituição, por sua vez, corresponde ao volume reinfundido no paciente. Nos sistemas de HDF *online* mais comumente utilizados na prática atual, o volume convectivo é determinado em função do fluxo sanguíneo, da fração de filtração e do tempo de tratamento.

A modalidade mais utilizada na Europa e no Brasil é a pós-dilucional^21,23,30–33^. Nessa estratégia, a solução de substituição é infundida após o dialisador, preservando concentrações mais elevadas de solutos no seu interior e proporcionando maior eficiência depurativa por unidade de volume convectivo, especialmente para pequenos solutos e moléculas médias. Em contrapartida, a progressiva hemoconcentração intradialisador favorece aumento da viscosidade sanguínea, deposição de proteínas na membrana, elevação da pressão transmembrana (PTM) e maior risco de coagulação do sistema. Por esse motivo, a fração de filtração (FF) costuma ser limitada a 25% do fluxo sanguíneo, podendo atingir valores próximos de 30% em condições técnicas adequadas^10,26,30^. A FF deve ser entendida como a proporção de fluido removido do plasma durante a passagem pelo dialisador, e não apenas do sangue total, aspecto particularmente relevante em pacientes com hematócrito mais elevado, nos quais a hemoconcentração, o aumento da PTM e as limitações na entrega da dose convectiva podem ser mais pronunciados. Para a realização de HDF pós-dilucional de alto volume, geralmente são necessários fluxo sanguíneo (FS) elevado, tipicamente superior a 350 mL/min, e acesso vascular bem funcionante^27^. Nessas condições, altos volumes convectivos são mais facilmente alcançados em pacientes com fístulas arteriovenosas, em comparação com cateteres venosos centrais. A dose convectiva nessa modalidade é influenciada principalmente pelo tempo de sessão, pelo FS e pela FF, sendo esta um dos principais fatores limitantes^10^.

Na modalidade pré-dilucional, a solução de substituição é infundida antes do dialisador, promovendo a hemodiluição do sangue que entra no dialisador. Essa configuração reduz a hemoconcentração intradialisador, minimiza a deposição proteica na membrana e atenua a elevação da PTM, favorecendo a realização da terapia mesmo em condições de menor FS. Em contrapartida, como a diluição reduz a concentração dos solutos à entrada do dialisador, a eficiência depurativa por litro de substituição é menor do que na modalidade pós-dilucional, exigindo maior volume de substituição para se obter uma dose convectiva equivalente. No Japão, onde são habitualmente empregados fluxos sanguíneos mais baixos, em torno de 250 mL/min, e dialisadores de alto fluxo^32^, a modalidade pré-dilucional é amplamente utilizada. Nessa modalidade, a ausência de hemoconcentração significativa reduz a limitação imposta pela FF, permitindo atingir a dose convectiva com menor dependência de FS elevados, embora ao custo de maior consumo de volume de substituição.

Em síntese, a HDF pós-dilucional apresenta maior eficiência depurativa por unidade de volume convectivo, porém depende de condições técnicas mais favoráveis, sobretudo de FS elevado e de acesso vascular de bom desempenho. Já a HDF pré-dilucional é menos eficiente por litro de substituição, mas oferece maior estabilidade operacional, menor hemoconcentração intradialisador e maior factibilidade em pacientes com limitações de acesso vascular ou de FS. De todo modo, a tolerância clínica e a efetividade global podem ser semelhantes entre as duas modalidades, desde que sejam respeitadas as particularidades técnicas de cada estratégia^31^. Assim, a escolha da modalidade deve ser individualizada, considerando os objetivos terapêuticos, o desempenho do acesso vascular, a capacidade de atingir o volume convectivo alvo e as características da membrana utilizada.

### Evidências científicas da HDF

#### Redução da mortalidade

A evidência sobre a HDF em pacientes com DRC-5D evoluiu de ensaios clínicos randomizados (ECRs) com resultados inicialmente heterogêneos para metanálises e estudos de vida real mais consistentes. Enquanto o estudo CONTRAST e o estudo turco não demonstraram redução significativa da mortalidade na análise principal, o ESHOL mostrou redução da mortalidade global com HDF pós-dilucional de alto volume convectivo, sugerindo que o benefício está relacionado à adequada entrega da dose convectiva^34–36^.

Esse achado foi reforçado por metanálises subsequentes. A metanálise de dados individuais publicada em 2016 demonstrou redução da mortalidade global e cardiovascular com HDF, com efeito mais evidente quando o volume convectivo excedia 23 L/sessão^37^. De forma consistente, o estudo CONVINCE confirmou redução de 23% na mortalidade por todas as causas com HDF de alto volume, além de menor mortalidade por infecção, em comparação com a HD de alto fluxo^21^.

Metanálises mais recentes consolidaram esses achados, confirmando redução da mortalidade global, cardiovascular e por infecção com HDF, além de relação dose-resposta entre maior volume convectivo e menor risco de óbito, especialmente com volumes ≥ 23 L/sessão^38,39^. No Brasil, esses resultados foram corroborados por uma coorte retrospectiva multicêntrica, na qual a HDF de alto volume se associou à redução da mortalidade global e cardiovascular/cerebrovascular^40^, resultado similar ao de outros estudos observacionais^41,42^.

#### Redução da taxa de hospitalização

Considerando a aplicabilidade da HDF na prática clínica fora do cenário de um ECR, encontramos evidências que corroboram seu benefício de mundo real. Um grande estudo internacional retrospectivo com 71.669 pacientes comparou HDF de alto volume com HD de alto fluxo e mostrou redução da taxa de internações [*incidence rate ratio* (IRR) 0,80; IC 95% 0,79–0,82] e dos dias de hospitalização (IRR 0,80; IC 95% 0,78–0,82), com consistência em diversos subgrupos clínicos e de prática dialítica^43^.

#### Benefício no acesso vascular

No eixo do acesso vascular, pilar das terapias extracorpóreas e, no caso da HDF, uma das principais variáveis para se atingir um volume convectivo adequado, um estudo recente de coorte japonesa demonstrou que a HDF se associou à maior perviedade primária da fístula aos 24 meses (74,0% vs. 48,7% em HD) e à redução significativa da perda de perviedade primária (HR 0,30; IC 95% 0,16–0,56), além de menor necessidade de intervenções endovasculares, mesmo após ajuste para variáveis de confusão^44^.

#### Benefício metabólico

Em relação ao impacto da HDF nos marcadores laboratoriais, uma redução significativa do fosfato sérico é relatada, especialmente quando volumes convectivos elevados são atingidos, o que pode diminuir a necessidade de quelantes de fósforo^45,46^. Em relação às toxinas urêmicas ligadas à proteína, como indoxil sulfato e p-cresil sulfato, a HDF mostrou-se mais eficaz na redução das concentrações pré-diálise desses marcadores, com efeito mais pronunciado para p-cresil sulfato em pacientes que atingem volumes convectivos superiores a 27,5 L^46^.

A HDF pós-dilucional remove ainda mais beta-2-microglobulina (β2M) do que a HD de alto fluxo^47,48^. O estudo de Armenta-Álvarez et al. demonstrou que a HDF proporciona maior taxa de remoção de β2M quando comparada à HD de alto fluxo, mantendo um perfil aceitável de perda dialítica de albumina sob parâmetros adequados de prescrição. O componente convectivo da HDF mostrou-se determinante para a superioridade na depuração de moléculas na faixa de peso molecular intermediário (~11,8 kDa), reforçando a plausibilidade fisiopatológica do benefício^49^.

#### Melhora dos sintomas e da qualidade de vida

A HDF tem sido associada à melhor tolerância ao tratamento e à melhora de sintomas relatados pelos pacientes quando comparada à HD de alto fluxo. A melhor tolerância hemodinâmica da HDF foi demonstrada de forma clara no estudo de Morena et al., no qual pacientes idosos apresentaram melhor tolerância ao tratamento quando comparados aos pacientes submetidos à HD de alto fluxo, com menos episódios de alteração de sinais vitais, redução expressiva dos sintomas no período intradialítico e melhores desfechos relacionados à qualidade de vida^50^.

Há vários relatos de efeitos positivos da HDF sobre a qualidade de vida, entre os quais se inclui o ensaio CONVINCE^21,51^. Em estudo conduzido por Aichi et al., avaliando pacientes em programa de HD que apresentavam baixos níveis de qualidade de vida relacionada à saúde (QVRS), a conversão para HDF esteve associada à melhora significativa nos escores de qualidade de vida e no estado nutricional^52^. Em revisão sistemática e metanálise publicada por Bossola et al., que avaliou o impacto da HDF sobre qualidade de vida, fadiga e tempo de recuperação pós-diálise, a modalidade foi associada a desfechos mais favoráveis quando comparada à HD convencional^34^. A análise agregada demonstrou redução da fadiga e encurtamento do tempo de recuperação após as sessões, além de melhora nos domínios de QVRS, sugerindo um benefício clínico consistente^53^.

Assim, a crescente incorporação da HDF na prática clínica impõe a necessidade de um posicionamento baseado em evidências que oriente sua indicação, seus requisitos técnicos e sua aplicação nos diferentes cenários assistenciais.

Trata-se de um documento institucional de caráter educativo e não normativo. O objetivo deste posicionamento é fornecer recomendações da SBN, baseadas nas melhores evidências e no consenso de especialistas, para o uso da HDF na diálise de manutenção no Brasil. Busca-se padronizar conceitos, reduzir a variabilidade da prática clínica e apoiar a tomada de decisão clínica e gerencial, incluindo: seleção e indicação de pacientes; parâmetros mínimos de prescrição e metas operacionais; requisitos técnicos e de segurança; e aspectos éticos, regulatórios, financeiros e de custo-efetividade, com foco na qualidade e na segurança do cuidado ao paciente.

## MÉTODOS

Este documento consiste em recomendações oficiais da SBN e foi elaborado por autores integrantes da Diretoria Nacional, do Departamento de Diálise, do Departamento de Nefrologia Pediátrica e do Comitê de Pacientes da SBN, além de um convidado externo com ampla experiência em HDF em cenário internacional.

O processo de elaboração envolveu discussões estruturadas entre os autores, com base na literatura científica disponível e em diretrizes internacionais. Em etapa subsequente, o documento foi encaminhado para revisão das Regionais ativas da SBN e submetido à consulta pública no site da SBN por um período de dez dias, divulgada nas páginas eletrônicas e redes sociais da SBN, permitindo a participação de profissionais, serviços e demais partes interessadas. O formulário disponibilizado na consulta pública apresentava as recomendações do documento e permitia resposta estruturada por meio das opções “concordo com o documento”, “concordo parcialmente com o documento” e “discordo do documento”, além de um campo aberto para comentários, justificativas e sugestões de modificação. As contribuições recebidas foram analisadas e sistematizadas pelo grupo, com incorporação das sugestões pertinentes e com justificativa quando não incorporadas, com o objetivo de maximizar a transparência, a legitimidade e a aderência às necessidades do país.

Após a consolidação pós-consulta pública, o documento passou por revisão editorial e aprovação final pelos responsáveis, resultando na versão definitiva das recomendações.

### Resultados da consulta pública

Na consulta pública, foram registradas 124 contribuições, das quais: 118 (95,2%) manifestaram concordância integral, 5 (4,0%) concordância parcial e 1 (0,8%) discordância. Das 124 contribuições, foram sugeridos cinco itens de modificação do documento, dos quais 4 (80%) foram incorporados à versão final do documento. Dos participantes da consulta pública, 74 (59,7%) eram nefrologistas, 30 (24,2%) pacientes, 14 (11,3%) profissionais de saúde e 5 (4,0%) demais partes interessadas. As contribuições da consulta pública, com as respectivas considerações dos autores, estão disponíveis por meio de solicitação à SBN e mediante justificativa razoável. Os nomes dos autores das contribuições na consulta pública foram retirados seguindo recomendação do Departamento Jurídico da SBN.

## DISCUSSÃO

### Elegibilidade do paciente para terapia de HDF

Como discutido anteriormente, a HDF associa-se à redução da mortalidade, com potenciais efeitos favoráveis também sobre as hospitalizações e a qualidade de vida. Sua indicação pode ser ampla em pacientes com DRC-5D, desde que haja decisão compartilhada e condições técnicas que permitam sua realização com segurança^38–40,43^.

Embora não existam contraindicações absolutas universalmente estabelecidas, a realização da HDF depende de condições clínicas apropriadas, infraestrutura adequada e capacidade de manter uma entrega convectiva efetiva. Além disso, seu efeito em comparação com a hemodiálise convencional não é homogêneo, podendo variar conforme o perfil clínico do paciente, as características do tratamento e a factibilidade de alcançar os parâmetros terapêuticos desejados^26^.

Essa heterogeneidade foi observada em um modelo preditivo derivado dos principais estudos comparativos entre HD e HDF, no qual se identificou tendência de maior ganho em sobrevida entre pacientes mais jovens, com menor carga de comorbidades, maiores níveis de albumina e creatinina e menores níveis de proteína C reativa^21^.

De modo geral, com base na plausibilidade clínica e na experiência acumulada, os benefícios da HDF podem ser mais evidentes: (1) em pacientes com menor função renal residual—que podem se beneficiar da ampliação do espectro de toxinas urêmicas removidas por uma técnica que combina difusão e convecção; e (2) em indivíduos mais jovens, com maior expectativa de vida e sem perspectiva de transplante renal—o que pode estar associado a uma maior exposição cumulativa à terapia e aos seus potenciais benefícios. Em cenários de limitação de recursos, esses perfis podem ser considerados prioritários para a incorporação da HDF^26^.

Assim, a indicação da HDF deve ser individualizada, considerando o potencial benefício clínico, a viabilidade técnica, a preferência do paciente e a disponibilidade estrutural do serviço. Não se propõem critérios rígidos e universalmente predefinidos para a seleção de pacientes, devendo essa decisão permanecer como prerrogativa do médico assistente, com base na avaliação clínica individualizada.

### Limitações do acesso vascular ou hemoconcentração

Um requisito fundamental para o sucesso da HDF é o acesso vascular adequado na modalidade de HDF pós-dilucional. Para atingir o volume convectivo de ≥ 23 L, é necessário um acesso vascular capaz de fornecer um FS superior a 350 mL/min^27,30^. A hemoconcentração também é relevante, pois, com hematócrito > 35%, o sangue torna-se mais viscoso, elevando a FF e podendo comprometer a entrega adequada da terapia^2^.

### Idosos frágeis, com comorbidades avançadas

Além da sobrevida, é importante ressaltar a evidência de desfechos centrados no paciente. Em idosos, nota-se melhor tolerância intradialítica e tendência à menor hospitalização com o uso de HDF^50^. Na análise específica de qualidade de vida do CONVINCE, pacientes em HDF apresentaram declínio mais lento nos componentes de saúde física (*p* = 0,035) e da função cognitiva (*p* = 0,031), com melhora global da qualidade de vida relacionada à saúde aos 30 meses (*p* = 0,006), reforçando um impacto clínico relevante para os desfechos reportados pelos pacientes^51^. Quando se prioriza a qualidade de vida, a HDF passa a ser uma modalidade de interesse na população de idosos frágeis.

### Gestantes

Até o momento, porém, não há estudos específicos que avaliem o uso de HDF na gestação. A literatura disponível enfatiza a intensificação da HD convencional—com aumento da frequência e da duração das sessões—estratégia associada a melhores desfechos maternos e fetais^54^. No entanto, algumas experiências internacionais e posicionamentos de sociedades científicas sugerem potencial benefício da técnica, com base na plausibilidade fisiológica, particularmente pela maior eficiência depurativa e melhor tolerância hemodinâmica. Nesse contexto, a Sociedade Chilena de Nefrologia recomenda a HDF como modalidade preferencial para gestantes em diálise. Ainda assim, a ausência de evidências específicas limita a generalização dessa recomendação, devendo a escolha da modalidade ser individualizada^55^.

### Crianças

A HDF na população pediátrica começou na década de 1970, em Estrasburgo, França, com Fischbach e colaboradores, utilizando inicialmente a metodologia pré-dilucional. Diferentemente dos adultos, a prescrição de HDF em pediatria deve ser normalizada pela área de superfície corporal. A meta de volume convectivo recomendada é de 13–15 L/m^2^ por sessão em modalidade pós-dilucional. Para atingir esses volumes, é essencial otimizar o FS para 5–8 mL/min/kg ou 150–250 mL/min/m^2^. A FF deve ser mantida entre 30% e 35% do fluxo sanguíneo estabelecido, para evitar hemoconcentração e coagulação do sistema^56^.

Quando o tratamento com HDF é iniciado, sugere-se uma taxa de FS inicial de 90 a 100 mL/min nas primeiras sessões, com incrementos de 10 mL/min/m^2^ por semana, até 200–250 mL/min/m^2^, conforme tolerado. Um desafio crítico é a limitação de equipamentos para crianças pequenas. Atualmente, não há máquinas de HDF aprovadas para pacientes com menos de 10 kg. O modelo Fresenius 6008, por exemplo, é indicado para crianças acima de 10–12 kg, com o uso de circuitos pediátricos, conforme especificado nos Quadros 1 e 2 ^56^.

**Quadro 1.** Especificações e requisitos para HDF pediátrica.

Categoria técnica	Especificações e requisitos para HDF pediátrica
**Peso mínimo**	**10 kg a 12 kg** (limite para a máquina Fresenius 6008 com circuito pediátrico). Outros modelos exigem pesos maiores: **> 15 kg** (5008); **> 25 kg** (Baxter Artis Physio); **30 kg** (Dialog iQ). Não há máquinas validadas para pacientes com peso inferior a 10 kg
**Máquina**	**Fresenius 6008** (atualmente a principal máquina disponível para pediatria nesta faixa de peso) **Fresenius 5008/S** (em descontinuação) **Baxter Artis Physio** **Braun Dialog iQ**
**Especificações do dialisador**	**Coeficiente de peneiramento (** *β* **2M):** > 0,6 **Área de superfície:** entre 100% e 120% da área de superfície corporal da criança **Diâmetro da fibra:** ≥ 200 *μ*m (para minimizar hemoconcentração)
**Linhas de HDF**	**Circuitos pediátricos específicos** (linhas de baixo volume para reduzir o volume extracorpóreo) e **linhas de infusão/substituição descartáveis** integradas ao sistema *online*.
**Tipo de Fluido**	**Solução de substituição *online*** **Qualidade**: água ultrapura, estéril e apirogênica **Pureza:** < 0,1 UFC/mL e endotoxinas < 0,03 EU/mL

**Quadro 2.** HDF pediátrica com o sistema 6008.

Categoria técnica	Especificações para o sistema 6008 CAREsystem Paed
**Peso mínimo**	**10 kg a 12 kg** (limite operacional seguro ao utilizar o circuito pediátrico específico)
**Máquina**	Fresenius 6008 (modelo sucessor da 5008, atualmente a única disponível com validação para crianças nessa faixa de peso)
**Configuração**	**6008 CAREsystem Paed**: Sistema que inclui o circuito extracorpóreo pediátrico de baixo volume para minimizar o risco de instabilidade hemodinâmica
**Especificações do dialisador**	**Alto fluxo** KUF: > 20 mL/h/mmHg (idealmente > 50 para máxima eficiência); permeabilidade para *β*2M > 0,6 Superfície: entre 100% e 120% da área de superfície corporal do paciente
**Linhas de HDF**	**Circuitos pediátricos integrados:** linhas de sangue de baixo volume morto e linhas de infusão para reposição *online* automatizada
**Tipo de fluido**	**Solução de substituição *online* (água ultrapura):** Padrão: estéril e apirogênica (filtração interna e retrofiltração protegida por filtros retentores de endotoxinas) Pureza: < 0,1 UFC/mL e endotoxinas < 0,03 EU/mL
**Modo de infusão**	**Pós-diluição**: modo preferencial com o uso do **AutoSub Plus™**, que otimiza automaticamente o volume convectivo e a fração de filtração em tempo real
**Pontos de atenção na prática clínica**	• Volume convectivo: a meta terapêutica deve ser de 12 a 15 L/m^2^ por sessão para garantir os benefícios para o crescimento e a redução da inflamação • Fração de filtração (FF): o sistema 6008 permite manter uma FF segura de até 30–35%, prevenindo a hemoconcentração excessiva no dialisador • BVM (monitor do volume de sangue): monitora variações do volume sanguíneo relativo durante a diálise • BTM (monitor da temperatura do sangue): monitora a temperatura do sangue e a recirculação do acesso vascular • Monitor OCM (*online clearance monitor*): monitora a eficácia da diálise (*clearance*) e a taxa de redução de ureia em tempo real • Acesso vascular: o sistema CAREsystem Paed exige fluxos sanguíneos entre 5 e 8 mL/min/kg para atingir a performance convectiva ideal

A HDF tem se destacado como uma modalidade potencialmente superior à HD convencional em crianças e adolescentes com DRC-5D, por sua capacidade de atenuar o risco cardiovascular, proporcionar melhor controle da pressão arterial, reduzir a concentração de toxinas inflamatórias, favorecer a saúde óssea e, sobretudo, melhorar a qualidade de vida, com menor tempo de recuperação pós-diálise, maior aptidão física e menor absenteísmo escolar^56–59^.

A doença cardiovascular subclínica é prevalente em crianças e adolescentes em diálise. O estudo observacional multicêntrico 3H *(The HDF, Heart and Height Study)* ^60^, de 2019, é o único estudo pediátrico multicêntrico envolvendo países europeus e da América do Norte que compara HDF e HD. Os dados do 3H sugerem benefícios significativos da modalidade HDF em relação à HD, após um ano de terapia. O estudo utilizou a modalidade pós-dilucional e demonstrou aumento significativo do escore Z da espessura médio-intimal da carótida (cIMT) em crianças em HD, enquanto esse parâmetro permaneceu estável no grupo submetido à HDF. Na análise por escore de propensão, a HD foi associada a um aumento de 0,47 no escore Z anualizado da cIMT em comparação com a HDF. O escore Z da pressão arterial média aumentou apenas no grupo HD. Os fatores associados a escores Z mais elevados de cIMT e de pressão arterial média foram o grupo HD, maior taxa de ultrafiltração e níveis mais elevados de β2M. O grupo HDF apresentou níveis mais baixos de β2M, paratormônio e proteína C reativa de alta sensibilidade^58,60,61^.

O retardo do crescimento é uma complicação impactante em crianças dialisadas. Fischbach e colaboradores foram os primeiros a demonstrar que o retardo do crescimento em crianças com DRC-5D poderia ser revertido por meio da HDF diária, com melhora significativa na velocidade de crescimento e no escore Z de altura, que aumentou no grupo HDF, mas permaneceu estável no grupo HD. Esse efeito benéfico é atribuído à depuração de citocinas inflamatórias e inibidores somatomedínicos (como a β2-microglobulina), o que ajuda a reduzir a resistência ao hormônio do crescimento. Além disso, observa-se melhora no apetite e no índice de massa corporal^62^.

A HDF melhora a saúde óssea ao favorecer um perfil osteoanabólico. A razão entre os marcadores de formação e reabsorção óssea aumenta em crianças em HDF, atingindo níveis comparáveis aos de crianças saudáveis. Outro benefício vital é a redução de 25% nos níveis de FGF23, uma toxina que, em excesso, está ligada à hipertrofia ventricular esquerda e à mortalidade. Em contrapartida, crianças em HD apresentam um aumento de mais de 100% dos níveis de FGF23 ao longo de um ano^63,64^.

Para adolescentes e crianças, a HDF traduz-se em uma vida mais ativa. Esses pacientes relatam menos sintomas intradialíticos, como redução de cefaleia, vertigem e cãibras. Além disso, esses pacientes têm menor tempo de recuperação pós-diálise, melhora na frequência às aulas e maior capacidade para atividades físicas^65^.

Acrescenta-se ainda que a HDF é bem tolerada, com estabilidade hemodinâmica e menos episódios de hipotensão intradialítica. A terapia não demonstra diferença significativa na perda da função renal residual em comparação com a HD e nem parece afetar negativamente a albumina sérica^63–65^.

Em conclusão, a HDF de alto volume desponta como a modalidade dialítica preferencial para crianças e adolescentes, por estar associada a melhores desfechos clínicos em comparação com a HD convencional, incluindo maior remoção de moléculas médias, redução do estado inflamatório crônico, impacto favorável sobre o crescimento estatural e a saúde óssea, maior estabilidade hemodinâmica e proteção vascular, promovendo melhor controle da pressão arterial, melhor qualidade de vida e maior participação social.

Recomendação da SBN: A HDF pode ser considerada para pacientes com DRC-5D, desde que haja decisão compartilhada, viabilidade técnica, acesso vascular adequado ou estratégia compatível com a modalidade escolhida, possibilidade de atingir uma dose convectiva efetiva e infraestrutura adequada do serviço. Em cenários de limitação de recursos, podem ser considerados prioritários crianças, pacientes com sintomas intradialíticos, aqueles com menor função renal residual ou maior carga de moléculas médias, pacientes jovens sem perspectiva de transplante renal, idosos frágeis com pior tolerância à HD convencional e pacientes com maior potencial de benefício clínico, sem que esses critérios configurem indicação obrigatória ou restritiva.

### Evidências em HDF domiciliar, incremental, mais frequente ou noturna

Até o momento, não há estudos robustos que comparem diretamente HDF domiciliar com HDF realizada em centro de diálise. A literatura aborda as seguintes comparações entre modalidades (HD vs. HDF) e locais (domiciliar vs. centros de diálise), conforme ilustrado no Quadro 3.

**Quadro 3.** Estudos comparativos entre HDF e HD domiciliar ou no centro de diálise.

Comparação	Evidência disponível	Tipo de estudos	Principais conclusões
**HDF domiciliar vs. HDF em centro**	Não	—	Não foram encontrados estudos randomizados ou de grandes coortes comparando diretamente a HDF nesses dois cenários.
**HDF em centro vs. HD em centro**	Sim	Ensaios clínicos e metanálises	HDF de alto volume associada a menor mortalidade e, possivelmente, a menos eventos cardiovasculares.
**HD domiciliar vs.** **HD em centro**	Sim	Coortes observacionais, revisões sistemáticas	Estudos sugerem melhor qualidade de vida, maior flexibilidade e, em alguns estudos, melhor sobrevida da hemodiálise domiciliar.
**Local de tratamento (domicílio vs. centro) nos estudos de HDF**	Não	—	Estudos de HDF não estratificam resultados por local de tratamento.

Abreviaturas – HD: hemodiálise; HDF: hemodiafiltração.

Embora não existam estudos comparando diretamente a HDF domiciliar com a realizada em centro de diálise, múltiplas evidências demonstram que a HDF de alto volume realizada em centro está associada à menor mortalidade no Brasil^40–42^ e no mundo^21,34–36^. Dessa forma, é plausível supor que parte dos benefícios fisiológicos da HDF também possa ser observada no ambiente domiciliar. No entanto, essa extrapolação deve ser interpretada com cautela, uma vez que ainda carece de confirmação por evidências específicas.

Não podemos, até o momento, concluir, por absoluta falta de dados, se a HDF domiciliar pode proporcionar uma redução adicional da mortalidade em comparação com a HDF realizada nos centros. Uma diálise mais frequente pode levar a uma melhor sobrevida; porém, não há comparação na literatura entre as modalidades de HDF e HD no que diz respeito à diálise mais frequente ou à diálise longa noturna.

Quanto à HDF incremental, não há evidências da sua superioridade em comparação com a HD incremental. É pouco provável, porém, que o benefício da HD incremental^66^ não seja observado também com a HDF. Em relação à HDF decremental, atualmente não há estudos específicos sobre o uso de HDF na função tardia do enxerto após transplante renal.

Em relação à HDF longa noturna realizada em dias alternados, Maduell F et al.^67^ demonstraram, em um estudo observacional de centro único, boa tolerância clínica e ausência de sintomas adversos, com melhora do controle do metabolismo cálcio-fósforo e da pressão arterial e redução do uso de quelantes de fósforo e de anti-hipertensivos. Um aspecto importante é que 62% dos pacientes mantiveram atividade profissional, dado semelhante ao encontrado em estudo observacional realizado no nosso meio^68^.

Recomendação da SBN: A HDF domiciliar, incremental, mais frequente ou noturna deve ser considerada apenas em serviços com estrutura técnica, capacitação da equipe, monitoramento, suporte assistencial e segurança compatíveis. Na ausência de evidências diretas robustas, a extrapolação dos benefícios da HDF de alto volume realizada em centro para esses cenários deve ser feita com cautela e com indivi­dualização da prescrição.

### Parâmetros mínimos sugeridos para a prescrição de HDF em adultos

A decisão entre HD convencional e HDF de alto volume deve ser compartilhada entre a equipe, o paciente e os familiares. É fundamental que o paciente compreenda os benefícios, riscos e exigências de cada método, favorecendo a adesão ao tratamento ao alinhar expectativas, especialmente quanto ao tempo de sessão, à necessidade de acesso vascular adequado e à assiduidade às sessões^69^.

### Transição da HD para HDF (abordagem *step-up*)

A migração da HD para HDF deve ocorrer após a otimização prévia da HD, permitindo ajustes progressivos, maior segurança e melhor eficácia terapêutica^32,70,71^.

Recomenda-se:

1. Uso de dialisadores de alto fluxo: coeficiente de ultrafiltração (KUF) > 50 mL/h/mmHg/m^2^ e coeficiente de peneiramento para β2M > 0,6.
2. Sessões de quatro horas, três vezes por semana, para a maioria dos pacientes.
3. Acesso vascular adequado: FS de 350–450 mL/min, com agulhas de calibre 14–15 gauge (G) em fístula ou enxerto, ou cateter ≥ 14 French (F) na modalidade pós-dilucional^72^.
4. A prescrição deve considerar o volume líquido de ultrafiltração necessário, buscando estabilidade hemodinâmica e tolerância clínica ao procedimento.
5. Prescrição progressiva: iniciar com FS de 250 mL/min por 15 dias, evoluir para 300 mL/min por mais 15 dias e, posteriormente, atingir o máximo efetivo. Essa estratégia pode não ser aplicável aos pacientes migrando da HD, já com FS elevado e previamente tolerado. Iniciar com volume convectivo de 15–18 L/sessão, com aumento gradual em 2 e 3 semanas, conforme tolerância clínica.

#### Componentes da prescrição de HDF^2,26^

A prescrição deve incluir parâmetros técnicos e operacionais mínimos bem definidos:

– A modalidade pós-dilucional é a mais estudada e geralmente preferencial, por concentrar a maior parte das evidências disponíveis. Exige menor volume de substituição, com menor consumo de recursos e maior sustentabilidade operacional. A pré-dilucional pode ser considerada em situações de limitação de FS (<350 mL/min) ou maior risco de hemoconcentração no filtro, como em pacientes com hematócrito elevado, necessidade de grande volume líquido de ultrafiltração, hipovolemia relativa, hipotensão intradialítica recorrente, baixo fluxo sanguíneo ou limitação de acesso vascular.
– A FAV é o acesso de escolha, seguida de enxerto e cateter tunelizado.
– Utilizar agulhas de calibre 14–15G para atingir FS > 350 mL/min; agulhas de calibre 16G podem ser utilizadas em casos selecionados de FS < 350 mL/min na pré-dilucional.
– Fluxo inadequado ou recirculação do acesso vascular contraindicam a HDF de alto volume pós-dilucional. Recomenda-se FS de 350–450 mL/min (pós) e 200–450 mL/min (pré).
– Duração mínima de quatro horas, três vezes por semana, para a maioria dos pacientes.
– Composição do dialisato semelhante à da HD, com fluxo de 400–500 mL/min. Em sistemas modernos de HDF *online*, o fluxo de dialisato (FD) pode ser ajustado em relação ao FS e ao fluxo de reposição. Quando mantido em torno de 20% acima do FS, pode preservar a depuração de pequenos solutos e favorecer maior entrega da dose convectiva, sem aumento proporcional do consumo de água ultrapura^2^. Não se recomenda o uso rotineiro de dialisato sem glicose ou com concentração de potássio abaixo de 2,0 mEq/L, pelo maior risco de eventos adversos, salvo em situações excepcionais e com monitoramento rigoroso.
– Na modalidade pós-dilucional, manter fração de filtração ≤ 30% do FS para evitar hemoconcentração, perda de albumina e coagulação do circuito^73^.
– Fluxo convectivo de 80–160 mL/min na modalidade pós-dilucional e 160–300 mL/min na modalidade pré-dilucional, com volume convectivo mínimo de ≥ 23 L/sessão ou ≥ 70 L/semana na pós-dilucional, e de ≥ 46 L/sessão ou ≥ 140 L/semana na pré-dilucional.
– Utilizar dialisadores com KUF > 50 mL/h/mmHg/m^2^, coeficiente de peneiramento para β2M > 0,6, baixa permeabilidade à albumina (*sieving* < 0,001) e superfície de 1,5–2,2 m^2^.
– Seguir protocolo institucional de anticoagulação, com possíveis ajustes na modalidade pós-dilucional para evitar coagulação do sistema.

O Quadro 4 resume as recomendações mínimas para a prescrição de HDF^10,74,75^.

**Quadro 4.** Recomendações mínimas para prescrição de HDF *online* de alto volume.

**Acesso vascular**	FAV (1^a^ opção)/enxerto/cateter tunelizado; evitar alto volume se fluxo inadequado/recirculação.
**Agulha**	14 ou 15G *→* FS > 350 mL/min; 16G *→* FS de 300 a 350 mL/min (casos selecionados).
**Fluxo de sangue (FS)**	Pós: 350–450 mL/min; Pré: 200–450 mL/min.
**Fluxo de dialisato (FD)**	400–500 mL/min.
**Tempo**	≥ 4 h/sessão (12–15 h/semana).
**Fração de filtração (FF)** (razão entre o volume convectivo e o FS)	≤ 30% (ideal ≤ 25%) Pós-dilucional.
**Modalidade**	Pós-dilucional (mais estudada); considerar pré-dilucional se FS < 350 mL/min.
**Fluxo convectivo**	Pós: 80–160 mL/min; Pré: 160–300 mL/min.
**Volume convectivo** (soma do volume de substituição e do volume de ultrafiltração)	Pós: ≥ 23 L/sessão ou ≥ 70 L/semana; Pré: ≥ 46 L/sessão ou ≥ 140 L/semana.
**Tipo de dialisador de HDF**	K_UF_ > 50 e CS para β^2^-microglobulina > 0,60; área de superfície de 1,5 a 2,2 m^2^ em adultos; CS para albumina < 0,001; KoA para ureia > 600 e baixa resistência interna ao fluxo de sangue.
**Anticoagulação**	Heparina (HNF/HBPM) conforme protocolo; pode exigir ajuste na modalidade pós-dilucional.
**Metas**	Volume convectivo ≥ 23 L/sessão; spKt/V > 1,2.
**Monitorização**	Revisão mensal da prescrição e do acesso.

Abreviaturas – CS: coeficiente de peneiramento (*sieving coefficient*); HNF: heparina não fracionada; HBPM: heparina de baixo peso molecular; K_UF_: coeficiente de ultrafiltração.

#### Metas operacionais

Incluem volume convectivo de ≥ 23 L/sessão (pós-dilucional), spKt/V > 1,2, redução da hipotensão intradialítica e menor interrupção das sessões^21^. Deve-se monitorar o acesso vascular, as pressões do circuito^76^, parâmetros clínicos e laboratoriais, como estado nutricional, eletrólitos, anemia, tolerância ao procedimento e qualidade de vida^77,78^.

Embora o volume convectivo de ≥ 23 L/sessão seja uma meta prática na HDF pós-dilucional, esse alvo pode ser insuficiente em indivíduos com maior superfície corporal, devendo-se considerar o ajuste pela área de superfície corporal (ASC), pela fórmula 23 L × ASC/1,73 m^2^, para evitar subentrega relativa da dose convectiva^2^. O uso de dialisadores de maior superfície pode ser considerado como estratégia para alcançar a meta de volume convectivo, quando clinicamente apropriado e respeitadas as condições técnicas e de segurança.

A prescrição deve ser individualizada, com reavaliação mensal conforme a resposta clínica, garantindo adequação da dose, frequência, duração do tratamento e indicação da HDF.

#### Requisitos técnicos mínimos e segurança

Para implementar e manter um programa de HDF *online* de alto volume, existem requisitos que devem ser respeitados, a saber^26^:

1. Dialisato composto de água ultrapura.
2. Máquinas de HDF *online.*
3. Controle rigoroso de qualidade, que inclui procedimentos de desinfecção relacionados às máquinas de HDF e substituição periódica dos ultrafiltros esterilizantes.
4. Dialisador adequado de HDF (descrito no Quadro 4).
5. Equipe de profissionais capacitados para realizar HDF.
6. Acesso vascular adequado (descrito no Quadro 4).
7. Boas práticas: monitorar sinais e sintomas dos pacientes, otimizar a anticoagulação, a adequação dialítica e a gestão de indicadores de qualidade.

A seguir, descrevem-se em maior detalhe estes requisitos.

Tratamento de água: Considerando que a solução de substituição precisa ser infundida no paciente durante a HDF (pré- ou pós-dialisador), é necessário que seja estéril, apirogênica e produzida e monitorada a um custo aceitável para o sistema de saúde^23,26,30^. No Brasil, a Resolução (RDC) n° 11, de 2014, da ANVISA, estabelece as Boas Práticas para serviços de diálise^79^ e sociedades científicas buscaram se expressar sobre os padrões esperados de qualidade da água para HDF^80,81^. Além disso, as normas ISO 23500-1:2019 e ISO 23500-3:2019 descrevem requisitos específicos para este processo e os padrões de qualidade da água esperados para terapias hemodepurativas^10,82,83^.

Na máquina de HDF, o fluido de substituição, que é infundido na linha de sangue (pré- ou pós-filtro de HDF), é produzido *online* por meio da ultrafiltração do dialisato ultrapuro por uma série de filtros esterilizantes (chamados de ultrafiltros). Estes ultrafiltros têm capacidade de adsorção de endotoxinas de 1:1.000.000 e, dessa forma, a interposição de dois filtros em sequência permite uma retenção de 1:10^-12^, configurando o quese convencionou chamar de “esterilização a frio”. O processo requer que a água ultrapura atenda a padrões internacionais de qualidade microbiológica e química, como os definidos pela *International Organization for Standardization* (ISO) e pela *Association for the Advancement of Medical Instrumentation* (AAMI), com limites rigorosos para bactérias (<0,1 UFC/mL) e endotoxinas (<0,03 UE/mL). Posteriormente, a água ultrapura é misturada aos concentrados para formar o dialisato, que passa por ultrafiltros validados para produzir o fluido de substituição estéril^26,30,84,85^.

Na HDF, deve-se realizar a desinfecção da máquina conforme as recomendações do fabricante (incluindo os ultrafiltros), com teste de integridade antes de cada sessão e substituição dos filtros nos intervalos recomendados^30^. Uma vez confirmada a conformidade da água tratada com os padrões da RDC nº 11/2014 e assegurado que a operação e o monitoramento do equipamento de HDF sejam realizados conforme as instruções do fabricante, não se justificam testes adicionais de rotina^23^.

A monitorização na HDF deve contemplar parâmetros essenciais para controle de eficácia e segurança nas terapias convectivas, incluindo fluxo sanguíneo (FS) e volume convectivo. Atingir volumes convectivos adequados em cada sessão constitui um elemento central da eficácia da HDF^21,75^. O prontuário deve permitir o registro sistemático desses parâmetros para gerar indicadores de seguimento e gestão, com destaque para a tolerância hemodinâmica e os eventos adversos^86^. A ANVISA orienta práticas de segurança do paciente em serviços de diálise, incluindo protocolos para qualidade e monitoramento da água, gerenciamento de tecnologias, prevenção de infecções e coleta e análise de indicadores de qualidade, recomendações que também se aplicam à HDF^87^.

Considerando a importância do acesso vascular para atingir o volume convectivo adequado, deve-se avaliá-lo longitudinalmente, sendo sugerido o uso de um indicador baseado na relação entre o fluxo de sangue (FS) e a pressão arterial (PA) do sistema de diálise (FS/PA), que reflete a patência do acesso vascular, cujo valor é usualmente de 2 mL/min/mmHg. Admite-se como um bom fluxo quando FS/PA > 1,5 mL/min/mmHg, ao passo que fluxos entre 0,8–1,5 mL/min/mmHg são tidos como ruins, e o acesso estaria em falência quando FS/PA < 0,8 mL/min/mmHg^26^. Quando o acesso vascular apresenta FS <350 mL/min, a causa do baixo fluxo deve ser investigada e tratada, podendo a HDF ser conduzida na modalidade pré-dilucional.

As modalidades pré- e pós-dilucional e os ultrafiltros estão exemplificados na Figura 1.

**Figura 1.** Esquema comparativo da HDF pós- e pré-dilucional, incluindo filtros, solução de substituição e água ultrapura.

Nota – Adaptado de Canziani MEF, et al.^23^

### Aspectos éticos, regulatórios, financeiros e de custo-efetividade

A implementação das recomendações deste documento deve considerar aspectos éticos, a capacidade dos sistemas de saúde de ofertar diálise e, no contexto brasileiro, possíveis ajustes regulatórios e o aperfeiçoamento dos modelos de remuneração. As decisões terapêuticas devem ser baseadas em informação clara e na decisão compartilhada; contudo, a assimetria de conhecimento entre profissionais e pacientes pode comprometer a autonomia decisória. Além disso, a indisponibilidade de determinadas modalidades terapêuticas pode limitar escolhas, agravar falhas de comunicação e restringir a autonomia de pacientes e profissionais^88^.

Os fatores éticos da coletividade estão relacionados com os preceitos de integralidade e justiça social e, portanto, com a premissa de utilização dos recursos públicos disponíveis na saúde da melhor maneira possível^89,90^. Estudos de microcusteio indicam que o valor do procedimento por paciente-mês em HDF é superior ao valor da HD convencional e este, superior ao valor da DP^91–93^. Essa diferença pode ser inferior a 15% quando se consideram somente os valores financeiros para produção dos insumos localmente (custo); porém, a inclusão de outros componentes de custo (frete e impostos de importação, p.ex.) pode alterar os valores comercializados (preço)^94,95^.

No caso da terapia renal substitutiva (TRS) e da incorporação de tecnologias de alto custo, um comitê formado por membros da *American Society of Nephrology*, *European Renal Association*, *European Dialysis and Transplant Association* e *International Society of Nephrology* (ASN, ERA-EDTA e ISN) tem reforçado que as políticas públicas em Nefrologia devem se pautar pela proporcionalidade de ações e investimentos em prevenção, tratamento conservador, métodos de diálise e transplante renal^96–98^. Essa proporcionalidade não tem sido observada na oferta de serviços pelo Sistema Único de Saúde (SUS) no Brasil, conforme demonstrado em estudos recentes^99,100^.

Há cerca de cinco anos, a HDF foi incluída no Rol de Procedimentos da ANS^101^. No entanto, essa inclusão não foi acompanhada de uma Diretriz de Utilização Técnica (DUT), o que significa que os planos privados de saúde deveriam fornecer o procedimento ao beneficiário, independentemente de critérios técnicos, como idade, comorbidades, exames complementares, entre outros.

No caso da TRS, em que o modelo de remuneração predominante no Brasil é o de pagamento por unidade de serviço, a ausência de DUT para HDF pode colaborar para negativas inadequadas por parte dos planos privados, assim como para solicitações desnecessárias por parte dos provedores de saúde^102,103^. A pactuação de modelos de remuneração inovadores poderá amenizar esses eventos à medida que agreguem elementos de valor em saúde ao pagamento (indicadores de qualidade assistencial, consultas em pronto-socorro, hospitalizações, entre outros)^104,105^.

No Brasil, o sistema de saúde caracteriza-se pela coexistência do SUS, de caráter público e universal, e do setor de saúde suplementar. O SUS, cronicamente subfinanciado, é responsável pela assistência exclusiva de cerca de 75% da população^106^. Considerando a assistência em Nefrologia, estudos têm demonstrado diferenças preocupantes entre os desfechos clínicos de brasileiros com e sem saúde suplementar^98,107^. A incorporação desigual de tecnologias entre o sistema público e o de saúde suplementar representa motivo de preocupação. Embora a HDF tenha sido incluída no Rol de Procedimentos da ANS desde 2021, essa modalidade ainda não foi incorporada ao SUS. Atualmente, cerca de 40,5% dos pacientes em diálise no setor suplementar realizam HDF, em comparação com aproximadamente 0,5% no SUS^20^, cenário que pode contribuir para a ampliação das iniquidades no sistema de saúde brasileiro, estruturado sobre o princípio da equidade.

A avaliação de tecnologias em saúde (ATS) é fragmentada no Brasil, contando com dois principais órgãos reguladores: no SUS, a CONITEC (Comissão Nacional de Incorporação de Tecnologias no SUS) e, na saúde suplementar, a COSAÚDE (Comissão de Atualização do Rol de Procedimentos e Eventos em Saúde Suplementar/ANS). Essa duplicidade constitui uma fragilidade regulatória que pode contribuir para processos de judicialização contra o SUS e para iniquidades assistenciais entre os brasileiros com e sem planos de saúde. Austrália, Reino Unido e Canadá são exemplos de países que adotaram uma agência única para avaliação de tecnologias em saúde^108^.

Em concordância com dados internacionais, estudos brasileiros têm demonstrado, de forma consistente, resultados da HDF superiores aos da HD^23,40–42,109^, embora avaliações econômicas completas ainda não tenham sido realizadas (impacto orçamentário para o SUS, custo-efetividade com dados primários nacionais e uso de metodologias que incorporem determinantes sociais e diferenças regionais)^110^.

### Recomendações da SBN para HDF *online* de alto volume

As principais recomendações da SBN para HDF *online* de alto volume estão resumidas no Quadro 5.

**Quadro 5.** Resumo das recomendações da SBN para HDF *online* de alto volume.

• Realizar decisão compartilhada com paciente e familiares.
• Considerar HDF para todos os pacientes, desde que seja factível atingir uma dose convectiva efetiva.
• Priorizar, em cenários de limitação de recursos, pacientes com sintomas intradialíticos, menor função renal residual, acúmulo de β2-microglobulina, pacientes jovens e sem perspectiva de transplante renal.
• Alguns perfis clínicos podem ser considerados prioritários para incorporação da terapia, sem que isso configure critério rígido ou obrigatório de indicação, que deve permanecer individualizada e sob responsabilidade do médico assistente.
• Otimizar previamente HD de alto fluxo e migrar para HDF, com incremento gradual do volume convectivo.
• Preferir a modalidade pós-dilucional; considerar pré-dilucional quando FS <350 mL/min ou quando houver limitação do acesso/hemoconcentração.
• Garantir tempo de HDF de quatro horas por sessão, três vezes por semana, para a maioria dos pacientes.
• Almejar volume convectivo ≥ 23 L/sessão ou ≥ 70 L/semana na modalidade pós-dilucional. Na modalidade pré-dilucional, considerar metas aproximadamente duas vezes maiores, como ≥ 46 L/sessão ou ≥ 140 L/semana. Priorizar FAV como acesso vascular
• Em pós-dilucional, almejar FS de 350–450 mL/min (agulhas de calibre 14–15G em FAV; cateter tunelizado ≥ 14 F, quando aplicável).
• Evitar HDF de alto volume com fluxo inadequado/recirculação.
• Utilizar dialisador apropriado para HDF.
• Seguir rigorosamente os ciclos de desinfecção da máquina e a substituição do ultrafiltro.
• Garantir dialisato de água ultrapura.
• Monitorar indicadores, incluindo a taxa de volume convectivo.
• Pediatria: prescrição normalizada por superfície corporal; metas convectivas e limites de FF ajustados; reconhecer limitações de equipamento em pacientes com peso inferior a 10 kg.

## CONCLUSÃO

A HDF *online* de alto volume consolida-se como modalidade dialítica avançada, com racional fisiopatológico consistente e evidência contemporânea de benefício clínico, sobretudo quando há entrega efetiva da dose convectiva (alvo ≥ 23 L/sessão na modalidade pós-dilucional). No Brasil, sua expansão recente e os dados nacionais de viabilidade e segurança reforçam a necessidade de padronização técnica, monitorização sistemática e governança da qualidade. Este posicionamento da SBN propõe parâmetros mínimos de prescrição, metas operacionais e requisitos de segurança, além de reconhecer desafios éticos, regulatórios e econômico-financeiros. Sua adoção deve ser responsável, baseada em decisão compartilhada, seleção adequada de pacientes e infraestrutura apta a sustentar um controle rigoroso de qualidade e de indicadores assistenciais, com foco em cuidado seguro, efetivo e orientado ao valor.

## Data Availability

Nenhum dado novo foi gerado ou analisado neste estudo.
